# The efficacy of cognitive stimulation, cognitive training, and cognitive rehabilitation for people living with dementia: a systematic review and meta-analysis

**DOI:** 10.1007/s11357-024-01400-z

**Published:** 2024-11-01

**Authors:** Alice Paggetti, Ylenia Druda, Francesco Sciancalepore, Francesco Della Gatta, Antonio Ancidoni, Nicoletta Locuratolo, Paola Piscopo, Luca Vignatelli, Luciano Sagliocca, Antonio Guaita, Piero Secreto, Andrea Stracciari, Paola Caffarra, Nicola Vanacore, Elisa Fabrizi, Eleonora Lacorte, the Italian Dementia Guideline Working Group, P. Caffarra, A. Guaita, P. Secreto, A. Stracciari, N. Vanacore, L. Sagliocca, L. Vignatelli, P. Battista, C. Borgarelli, C. Cerami, A. Chiari, M. Corbo, A. Costa, M. Cotelli, A. Dodich, E. Faè, E. Favaretto, V. Frisardi, L. Fubini, M. Gandelli, D. Gollin, D. Imperiale, C. Ivaldi, F. Izzicupo, A. Lanzoni, V. Lavermicocca, M. Lazzarino, R. Liperoti, M. Massaia, G. Menabue, M. A. Molinari, A. Morelli, A. Peruzzi, F. Piazza, M. Possenti, C. Ricci, S. Russo, P. Spadin, G. Spalletta, D. Zenaro, A Ancidoni, F Della Gatta, E Fabrizi, E Lacorte, N Locuratolo, P. Piscopo, D. Coclite, A. Fauci, A. Napoletano, I. Bacigalupo, G. Bellomo, M. Canevelli, E. Carbone, A. Crestini, E. Distaso, Y. Druda, S. Esposito, G. Fumagalli, S. Morelli, A. Paggetti, G. Remoli, R. Rivabene, S. Salemme, F. Sciancalepore, M. Valletta, N. Veronese, V. Zaccaria, C. Bini, F. V. Mennini, P. Sciattella, A. Ceccarini, Z. Mitrova, S. Pizzarelli, R. Saulle, S. Gainotti, L. Riva, C. Petrini, S. De Masi, F. Nonino, G. Martelli, M. C. Porrello

**Affiliations:** 1 National Centre for Disease Prevention and Health Promotion, Italian National Institute of Health, Rome, Italy; 2 Department of Medical and Surgical Sciences, University of Bologna, Bologna, Italy; 3 Department of Human Neuroscience, Sapienza University of Rome, Rome, Italy; 4 Department of Neuroscience, Mental Health and Sense Organs (NESMOS), Faculty of Medicine and Psychology, Sapienza University of Rome, Rome, Italy; 5 Department of Neuroscience, Italian National Institute of Health, Rome, Italy; 6 IRCCS Istituto Delle Scienze Neurologiche Di Bologna, Bologna, Italy; 7 Local Health Unit, Salerno, Italy; 8 Golgi Cenci Foundation, Abbiategrasso, Milan Italy; 9 Alzheimer Unit, Fatebenefratelli Hospital, San Maurizio Canavese, (TO) Italy; 10 Cognitive Disorder Center, Neurology Unit, S.Orsola-Malpighi University Hospital, Bologna, Italy; 11 Dementia Unit AOU, Parma, Italy

**Keywords:** Dementia, Cognitive interventions, Guideline, Cognitive stimulation, Cognitive training, Cognitive rehabilitation, Systematic review

## Abstract

Cognition-oriented treatments (COTs) are a group of non-pharmacological treatments aimed at maintaining or improving cognitive functioning. Specific recommendations on the use of these interventions in people living with dementia (PLwD) are included in the Italian Guideline on the Diagnosis and Treatment of Dementia and Mild Cognitive Impairment, developed by the Italian National Institute of Health. This systematic review and meta-analysis, based on the GRADE methodology, is part of the guideline. Considered outcomes included the cognitive functions, quality of life, and functional abilities of PLwD, taking into account disease severity, modality and system of delivery, and form of the intervention. The effectiveness of these interventions on caregivers’ outcomes was also assessed. Both group and individual cognitive stimulation were reported as effective in supporting cognitive functions in PLwD at any degree of severity. Individual cognitive training and group cognitive training were reported as effective in improving global cognitive functions in people with mild dementia. Cognitive rehabilitation appeared to be effective only in improving the functional abilities of people with mild dementia. Cognitive rehabilitation appeared to be the most effective in improving caregivers’ outcomes, with results suggesting a reduction in care burden. The observed differences in the effectiveness of these interventions in people with different disease severity can be explained by the intrinsic characteristics of each intervention. Despite the large number of available studies, a high clinical, statistical, and methodological heterogeneity was observed. More methodologically rigorous studies are needed to clarify the effectiveness of each protocol and modality of intervention.

**Supplementary Information:**

The online version contains supplementary material available at 10.1007/s11357-024-01400-z.

## Introduction

Dementia is considered worldwide as one of the main causes of disability among older adults. Available estimates suggest that more than 55 million people are currently living with dementia (World Health Organization, [96]). People living with dementia (PLwD) often need to rely on care provided by either formal or informal caregivers, including family members, who are therefore exposed to a higher risk of various degrees of not only physical, but also emotional, psychological, social, and financial burden [38, 41]. Dementia thus affects the quality of life and well-being of both people living with it and their caregivers, thus highlighting the importance of a holistic approach to care to reduce as much as possible its impact on the everyday life of PLwD and their caregivers [91]. Non-pharmacological interventions (NPIs), sharing this approach, target a wide range of clinical outcomes, are usually non-invasive, and are less frequently associated with adverse effects compared to pharmacological treatments [6, 19]. Although some NPIs are also aimed at improving emotional and behavioral symptoms, there is a group of interventions called “Cognition-oriented treatments” (COTs), in which cognitive stimulation (CS), cognitive training (CT), and cognitive rehabilitation (CR) are commonly included. These interventions, though sharing some common characteristics, are defined as having different characteristics in literature. The Guideline on the assessment, management, and support of people with dementia (NICE Guideline 97, NG97), published in [69] by the National Institute for Health and Care Excellence (NICE), described these interventions as having different goals and modalities (NG97, [69]):

- CS targets involving people in a range of activities and discussions aimed at generally improving their cognitive and social functioning. It can be performed as individual or, more often, group sessions. Different forms are available based on different protocols such as reality orientation therapy (ROT) [85] and cognitive stimulation therapy (CST) [87, 95]. ROT can be easily implemented also in non-clinical settings and provided by caregivers at home. Differently, CST [87, 95] is a form of CS characterized by a defined intervention protocol and combines various elements and techniques (reality orientation, reminiscence, multisensory stimulation, and implicit learning) [18].
- CT is a guided practice on a set of standard tasks designed to reflect specific cognitive functions. CT can be designed to train either a single cognitive function or multiple cognitive domains simultaneously. It can be performed as an individual or group session. However, personalizing the intervention can be easier when implementing it in individual computerized sessions [6].
- CR is an individualized approach based on identifying goals that are relevant to the patient and working with them and their caregivers to achieve them. The approach focuses on improving or maintaining performance in everyday life and supporting independence. CR does not specifically aim to improve cognition but addresses the disability resulting from the impact of cognitive impairment on everyday functioning and activities (NG97, [69]). Evidence on the use of CR in people with dementia is still inconsistent and heterogeneous [13]. CR often requires involving caregivers in supporting people with dementia in implementing the intervention.

Despite the different definitions and theoretical basis, these three interventions are often combined, and sometimes, the terms defining them are applied interchangeably, making it difficult to clearly discriminate the individual contribution of each intervention [5, 6, 27, 39, 42, 92]. Moreover, COTs are often offered in combination with other NPIs, based on a multi-component approach [94]. For this reason, examining the contribution of each COT separately is crucial when investigating the effectiveness of these interventions on cognitive functions and functional abilities in PLwD. This was the main objective of this systematic review and meta-analysis, which was performed within the framework of the activities carried out for the development of the Italian guideline on the diagnosis and treatment of dementia and mild cognitive impairment (MCI) that was published in January 2024 by the Italian National Institute of Health (Istituto Superiore di Sanità, ISS) (ISS, [50]). The ISS was entrusted by the Italian Ministry of Health, as part of the activities of the 2021 Italian National Fund for Alzheimer’s and other dementias, developing a national guideline following the methodology standards of the Italian National Guideline System (Sistema Nazionale Linee Guida, SNLG).

Based on the SNLG Methodological Handbook (SNLG, [83]), the working group agreed to adapt and update the NG97. We hereby report and discuss the summary of evidence and metanalyses about the efficacy of CS, CT, and CR on cognitive functioning, functional abilities, and quality of life (QoL) of PLwD. We further analyzed and discussed, as a narrative summary, the comparative effectiveness of different CS (ROT, CST, and other CS protocols) and CT (traditional vs. computerized) protocols. We also focused on the role of caregivers, who often experience a significant care-induced burden, such as depressive symptoms and other neuropsychiatric sequelae. Specifically, we examined the indirect role of COTs on caregivers’ burden and quality of life.

## Materials and methods

This systematic review and meta-analysis is part of the activities carried out by the ISS to develop the national guideline “Diagnosis and treatment of dementia and Mild Cognitive Impairment.” All systematic reviews and meta-analyses in the guideline were performed according to the methodology described in the *Cochrane Handbook for Systematic Reviews* [47] and were reported based on the PRISMA (Preferred Reporting Items for Systematic Reviews and Meta-Analysis) statement for reporting systematic reviews and meta-analyses [76]. Evidence for all questions included in the guideline was analyzed based on the GRADE approach. When possible, meta-analyses were performed using the software Review Manager version 5.4. Forest plots were produced to provide a visual summary of data from included studies and the cumulative weighted estimate from all included studies. Forest plots included mean differences in the outcome measured from baseline to endpoint for each included study, along with their confidence intervals as a measure for the precision of the estimate, and the pooled estimate from the meta-analysis, weighted by the variances for each study, along with its confidence interval, as a measure for the cumulative effect of the intervention on the considered outcome. Results from the meta-analyses, being based on pooling results from all included primary studies, provide a more powerful estimate of the true effect size than those derived from a single study and increase the power and precision of estimates of treatment [59].

Further details on the methodology adopted for this systematic review and all the systematic reviews performed are reported in the full guideline (ISS, [50]). A visual representation of the process is reported in the flow diagram in Fig. S1.

### Information sources

The search strategy for questions on non-pharmacological interventions for people with dementia (Questions 20a, b, c, and d) in the Italian guideline was performed by adopting the search terms from the NG97 (Document S1). Bibliographic searches were carried out in December 2023 on the following databases: Cochrane Library, PubMed, and EMBASE.

### Eligibility criteria

Only randomized clinical trials meeting the following criteria were included:

Type of participants: People aged ≥ 40 years living with dementia.

Type of intervention: CS, CT, or CR, defined as reported above, either in individual or group session.

Type of comparators: Standard care.

Type of outcomes: Global cognitive functions, activities of daily living (ADL), and quality of life (QoL).

Articles not published in English were removed.

### Selection of sources of evidence and data charting process

The lists of records identified from the search strategy were uploaded on “Rayyan,” a web-based tool to manage the literature selection process [https://www.rayyan.ai/]. After removing duplicates, titles and abstracts were initially screened and selected based on their pertinence and relevance to the topic of the review by three couples of independent reviewers. Potential conflicts and disagreements were resolved by consensus. The full text of selected studies was retrieved, and they were applied to the predefined eligibility criteria. Systematic reviews were excluded but were considered separately to check the completeness and consistency of results.

### Data extraction and quality assessment

Data from the included studies were extracted using standardized forms. Extracted data included the following information: bibliographic reference, eligibility criteria, characteristics of participants, number of participants, type of intervention, definition of usual care, length of intervention, length of follow-up, and attrition. The interventions adopted in each of the included studies were described reporting the following information: intervention modality (individual or group session), system for delivering the exercises (computerized versus traditional, including paper and pencil or verbal exercises), and the form of the intervention. CS was classified as CST, ROT, or CSP (cognitive stimulation program), which was considered to cover all CS interventions that did not belong to either ROT or CST. CSP interventions were classified as multi-component when CSP was combined with other interventions (e.g., physical exercise, occupational therapy) that were also not performed by the control group; it was classified as a single component in all other cases. CT and CR were also classified as either single or multi-component.

Uncertainties and disagreements in data extraction and classification were resolved by involving other researchers in the discussion.

The quality assessment of the studies was performed using the Cochrane risk-of-bias tool (RoB) (J. P. T. [46]). The RoB tool is designed to assess the methodological quality of randomized trials by analyzing the potential for bias in six domains: (1) random sequence generation (selection bias), (2) allocation concealment (selection bias), (3) blinding of participants and personnel (performance bias), (4) blinding of outcome assessment (detection bias), (5) incomplete outcome data (attrition bias), and (6) selective reporting (reporting bias). Each domain can be rated as being at a low, unclear, or high risk of bias.

The overall assessment of evidence for each outcome was performed based on the GRADE approach. The certainty of evidence for each outcome was graded as very low, low, moderate or high.

### Data analysis

Meta-analyses were performed using the software Review Manager version 5.4. Analyses for each outcome of interest (cognitive functioning, QoL, and functional abilities) were performed separately for intervention modality and were stratified for dementia severity (mild, mild to moderate, and moderate).

Results from single studies were combined using meta-analyses. Pooled estimates were calculated along with their 95% confidence interval (CI) using a random effect model. Estimates of the effect of the intervention for each study were calculated using the mean change from baseline to the longest available follow-up. The combined estimate of the effect of the intervention was calculated as the weighted average of the estimates from individual studies. Results were reported as mean differences (MDs), when pooled studies adopted the same outcome measure, or as standardized mean differences (SMDs), when pooled studies adopted different outcome measures. Consistency of results was analyzed by considering it as the degree of homogeneity in the direction and size of the effect of an intervention on specific outcomes (Institute of Medicine (US) Committee on Standards for Systematic Reviews of Comparative Effectiveness Research et al., [49]). Heterogeneity among studies may be statistical (e.g., precision and direction of the estimates), clinical (e.g., characteristics of study participants or interventions), or methodological (e.g., study design). Heterogeneity was assessed using the Chi^2^ and *l*^2^ tests. Statistical significance for the Chi^2^ test was set at a *p* value of 0.1, and the *l*^2^ test was considered suggesting significant heterogeneity when values were ≥ 40% [47]. As relying only on statistics to test for and explore heterogeneity may be partial, clinical and methodological heterogeneity was also discussed in a narrative way (Institute of Medicine (US) Committee on Standards for Systematic Reviews of Comparative Effectiveness Research et al., [49]).

In case of heterogeneity across studies potentially reflecting differences in a treatment’s effect based on clinical differences, this was discussed to understand and further characterize those differences, which can have relevant implications in clinical practice (Institute of Medicine (US) Committee on Standards for Systematic Reviews of Comparative Effectiveness Research et al., [49]). Methodological heterogeneity due to biases in study design or execution was explored using methodological checklists and was discussed.

Subgroup analyses were also considered to explore heterogeneity. However, they were considered unfeasible and unreliable as the resulting subgroups were too small, and clinical heterogeneity was too high. As an example, when attempting to stratify studies according to the specific type of intervention adopted, the resulting subgroups were too small, and in some strata, most studies adopted the same type of intervention. When considering stratifying according to the length of the intervention and its intensity, the resulting subgroups were still heterogeneous in terms of intensity when stratifying for length (e.g., studies adopting interventions that had the same length but different intensity) and in terms of length when stratifying for intensity (e.g., studies adopting interventions that had the same intensity but different length). On this basis, heterogeneity was accounted for and discussed in the narrative summary of results.

Similarly, when considering the short-term and long-term effects of these interventions, no stratified analysis could be made, as studies either reported the effect of long-term interventions (e.g., lasting > 12 weeks with outcomes measured at different times during the intervention and immediately after its end) or the effect of short-term interventions over a long-term follow-up (e.g., lasting ≤ 12 weeks with outcomes measured at different times during the intervention, immediately after its end, and at different times of follow-up).

## Results

**Fig. 1:**
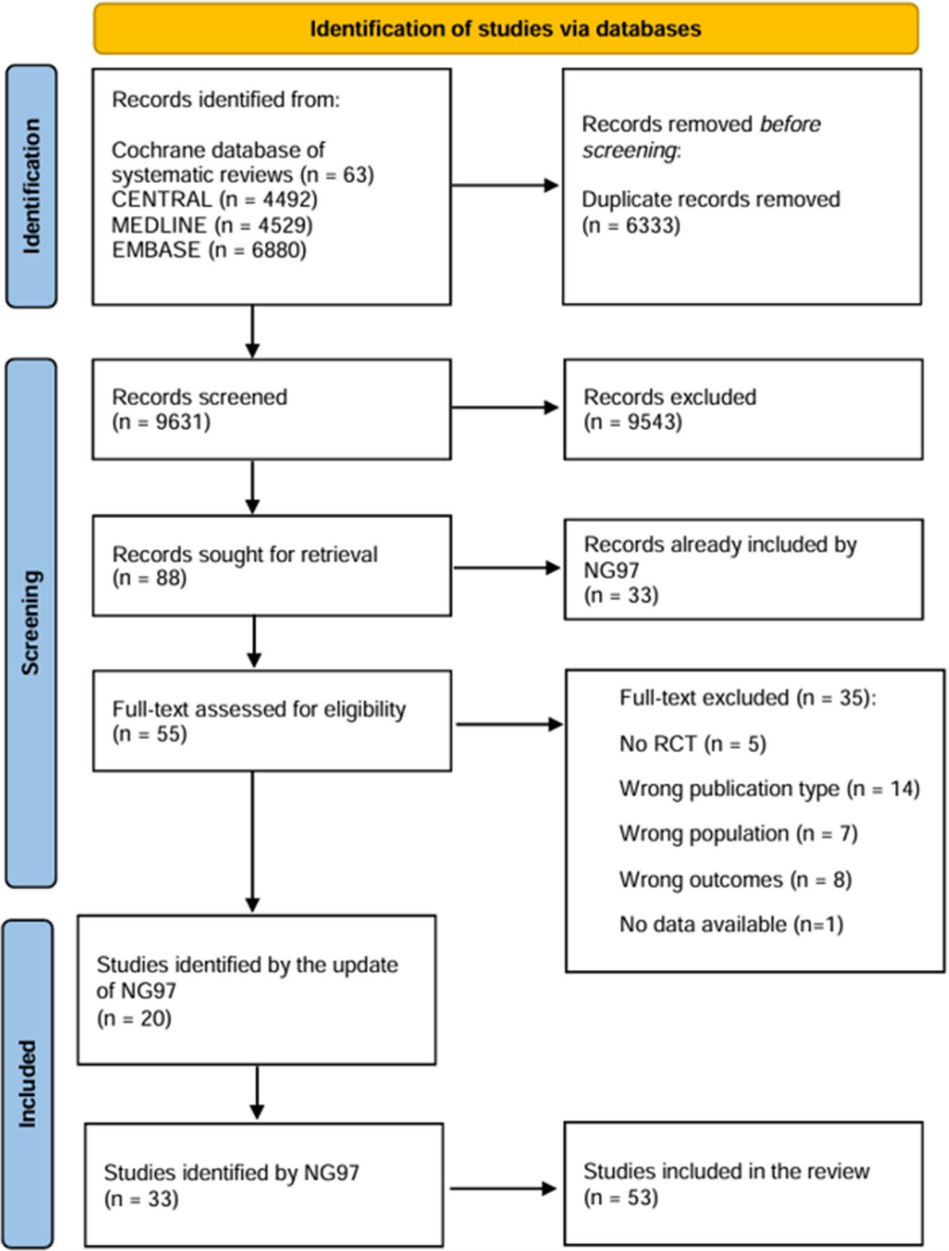
Flow diagram of the literature

Bibliographic searches on literature databases yielded 15,964 records. After removing duplicates, 6333 duplicates were excluded, and the remaining 9631 records were screened. Eighty-eight records were selected and retrieved in full text. A total of 53 trials met the inclusion criteria and were included. The characteristics of the included studies are reported in Table S1 and Table S2 in Supplementary Material. Of the 53 included trials, 33 were included by the NG97, while 20 were identified after its update. Overall, 29 trials investigated the use of CS [2, 3, 7, 8, 10–12, 14, 16, 17, 23, 29, 30, 35, 52, 53],H. J. [56, 66, 68, 70, 71, 73, 74, 78, 79, 86, 87, 98, 100], 17 the use of CT [9, 15, 20, 31–33, 37, 45, 54, 55, 58, 61, 64, 81, 88, 89, 99], and 5 the use of CR [13, 25, 26, 28, 57]. Tsantali et al. [90] investigated the use of both CS and CT, while Amieva et al. [4] investigated the use of both CR and CT. Information on the categorization and characteristics of the interventions is reported in Table 1. The date of publication of the studies ranged from 1991 to 2023. The flow diagram of the literature is reported in Fig. 1.

**Table 1:** Interventions characteristics of the included studies

Cognitive stimulation studies					
Reference	Description	Modality	Form	Multi-component	CS type
Alvares-Pereira et al. [2]	CST: sessions are mainly characterized by spatial and temporal orientation; main activity connected in each session to a different theme (e.g., physical games, word association, or number games)	Group	Traditional	No	Single-component CST
Alves et al. [3]	CSP: the sessions included various activities (e.g., identifying and categorizing objects, discussing about autobiographical past experiences) for stimulating different cognitive functions such as memory, language, attention, and reasoning	Group	Traditional	No	Single-component CSP
Baldelli et al. [8]	Formal ROT	Group	Traditional	No	ROT
Baldelli et al. [7]	Formal ROT and physical therapy program	Group	Traditional	Physical therapy program	ROT
Bottino et al. [11]	Cognitive rehabilitation training program: the intervention first included a temporal and spatial orientation, and then, cognitive activities were carried out. Patients shared life experiences and were trained on attention and memory strategies. The language was training discussing interesting themes in a group. ADLs were trained with simulation of activities of daily living. Moreover, patients did several external activities to stimulate social relationships. The use of external memory aid was trained at every session and at home with caregivers	Group	Traditional	Yes: elements of CT and CR	Multi-component CSP
Breuil et al. [12]	CSP: the sessions were divided into two parts. First part of the session: draw, recall words related to the drawing, and discuss topics related to the drawing made. Second part of the session: recognize, name, classify objects, and recall objects belonging to the same category, as well as recall topics related	Group	Traditional	No	Sigle-component CSP
Buschert et al. [14]	Multi-component cognitive intervention with emphasis on cognitive stimulation, including exercises for specific cognitive functions (e.g., memory, executive function, and attention), teaching and practicing of mnemonic techniques and strategies, education about age-associated changes, psycho-motor and recreational exercises, and exercises to stimulate cognitive interaction	Group	Traditional	Yes: elements of CT	Multi-component CSP
Camargo et al. [16]	Formal ROT (conducted by therapists) and informal ROT (caregivers were asked to stimulate patients daily)	Individual	Traditional	No	ROT
Capotosto et al. [17]	CST: sessions mainly characterized by spatial and temporal orientation; main activity connected in each session to a different theme (e.g., food, sounds, life)	Group	Traditional	No	CST
Chapman et al. [23]	CSP enhancing various cognitive abilities: verbal content through discussion of topics and narration of important life events, functional abilities by highlighting involvement in hobbies and activities at home or outdoors, and quality of life through educating participants and their families about dementia and advances in research	Group	Conversational interaction	No	Single-component CSP
Coen et al. [29]	CST: sessions mainly characterized by spatial and temporal orientation; main activity connected in each session to a different theme (e.g., food, sounds, physical games)	Group	Traditional	No	CST
Cove et al. [30]	CST: the intervention first included a temporal and spatial orientation, followed by a warm-up exercise and discussion of a recent news article. Moreover, participants did a main activity connected to different themes	Group	Traditional	No	CST
Juárez-Cedillo et al. [52]	CSP: each session included orientation to reality, cognitive stimulation of cognitive function (attention, memory, and language exercises), memory techniques (sharing personal experience), and an explanation of daily activities carried out at home. The activities cover cognitive, behavioral (i.e., promoting personal interaction), and psychological (i.e., increasing self-esteem and personal security) activities	Group	Traditional	Yes: elements of CT and CR	Multi-component CSP
Justo-Henriques et al. [53]	CST: sessions mainly characterized by spatial and temporal orientation; main activity connected in each session to a different theme (e.g. food, sounds, everyday life)	Individual	Traditional	No	CST
Kim et al. [56]	Multidomain CSP: including art therapy, music therapy, recollection therapy, horticultural therapy, physical exercise, and occupational therapy	Group	Traditional	Yes: art therapy, music therapy, recollection therapy, horticultural therapy, physical exercise, and occupational therapy	Multi-component CSP
López [66]	CSP: the intervention first included a temporal and spatial orientation, and then, cognitive activities were carried out. The sessions were focused on memory and gnosis, praxis and language, executive function, and calculation	Group	Traditional	No	Single-component CSP
Mapelli et al. [68]	CSP including personal, spatial, and temporal orientation and proceeding with structured stimulation for the cognitive domain, consisting of exercises specific for 5 areas (memory, language spatial and temporal orientation, attention, and logic)	Group	Traditional	No	Single-component CSP
Oliveira et al. [70]	CSP where the exercises were connected with instrumental activities of daily living (e.g., morning hygiene, grocery shopping, or choosing clothes)	Individual	Computerized cognitive program with non-immersive virtual reality	Yes: elements of CR	Multi-component CSP
Onder et al. [71]	Formal ROT (conducted by trained caregivers) and informal ROT (caregivers were asked to stimulate patients daily)	Individual	Traditional	No	ROT
Orgeta et al. [73]	CST: the program consisted of various themed activity sessions (e.g., being creative, number games, or art discussion) conducted by trained caregivers	Individual	Traditional	No	CST
Orrell et al. [74]	Maintenance CST: each maintenance session had a specific theme (e.g., current affairs, my life, word games) including orientation-based activity	Group	Traditional	No	CST
Requena et al. [78]	CSP comprising severel areas of stimulation (i.e., orientation, bodily awareness, family and society, caring for oneself, reminiscing, household activities and animals, people, and things). Visual stimuli were shown, and several questions related to the images were asked. Brief muscle relaxation preceded and followed each session	Group	Visual stimuli displayed on a television screen	No	Single-component CSP
Requena et al. [79]	CSP comprising severel areas of stimulation (i.e., orientation, bodily awareness, family and society, caring for oneself, reminiscing, household activities and animals, people, and things). Visual stimuli were shown, and several questions related to the images were asked. Brief muscle relaxation preceded and followed each session	Group	Visual stimuli displayed on a television screen	No	Single-component CSP
Spector et al. [86]	CST including sessions designed with 4 phases (i.e., the senses, remembering the past, people and objects, everyday practical issues)	Group	Traditional	No	CST
Spector et al. [87]	CST: sessions mainly characterized by spatial and temporal orientation; main activities connected in each session to a different theme (e.g., food, sounds, physical games). The final part of the sessions was dedicated to a warm-up activity	Group	Traditional	No	CST
Tsantali et al. [90]	CSP that included cognitive tasks that were not targeted a specific cognitive ability (e.g., drawing, painting, puzzles, copying a figure, looking and naming images, listening to music and singing, and having free discussion with the clinician)	Individual	Traditional	No	Single-component CSP
Yamanaka et al. [98]	CST: sessions mainly characterized by spatial and temporal orientation; main activity connected in each session to a different theme (e.g., food, sounds, physical games)	Group	Traditional	No	CST
Young et al. [100]	CST and tai chi. CST sessions were mainly characterized by spatial and temporal orientation; main activity connected in each session to a different theme (e.g. food, sounds, childhood)	Group	Traditional	Tai chi	CST
Cognitive training studies					
Reference	Description	Modality	Form	Multi-component	CS type
Amieva et al. [4]	Set of standard exercises involving various cognitive functions (memory, attention, language, and executive functions) organized in two levels of difficulties and designed to tap a specific activity of daily life	Group	Traditional	No	Single-component TCT
Bergamaschi et al. [9]	Specific tasks designed to stimulate various functions: spatial and time orientation, logical reasoning, memory, attention, perception, visual analysis, and recognition of emotional expressions (i.e., for time orientation: recognition and naming of images of clothing and association clothing with images of the four seasons). The difficulty increased gradually	Group	Traditional	No	Single-component TCT
Cahn-Weiner et al. [15]	Memory training intervention of the ACTIVE study: instruction and extensive practice in mnemonic strategies (categorization and visualization). It includes repeated presentation of the world list followed by recall and recognition tasks	Group	Traditional	No	Single-component TCT
Cavallo and Angilletta [20]	Cognitive training with Brainer© software which increases difficulties as performance improves. It involves various cognitive domains (visual perception, auditory perception, attention, language, reading, writing, calculations, logic, deduction, memory, and sensory-motor skills). Each treatment session involved one exercise per cognitive domain in random order	Individual	Computerized	No	Single-component CCT
Davis et al. [31]	In-home attention exercises directed by caregivers: space retrieval, peg task (presentation of rhyming number-object pairs), face-name training task, and cognitive stimulation (which involved exercises to stimulate the five attention levels of the APT Program, namely, focused, sustained, selective, alerting, and divided attention)	Individual	Traditional	Yes: elements of CS	Multi-component TCT
De Luca et al. [32]	Cognitive training involving various domains: attention (selective and divided), visual-spatial memory, verbal fluencies (phonemic and semantic), and praxis abilities (ideo-motor and constructive praxis). Tasks involved increasing difficulty (12 levels)	Individual	Computerized	No	Single-component TCT
De Vreese [33]	Cognitive training targeting memory, language, and executive function, with home practice facilitated by carers	Individual	Traditional	No	Single-component TCT
Galante [37]	Cognitive training exercises selected from the TNP software, involving various cognitive domains: memory (shopping list), language (denomination), perception, intelligence, attention, memory, and spatial cognition	Individual	Computerized	No	Single-component CCT
Heiss et al. [45]	Cognitive training consisting of solving perceptual, memory, or motor tasks which vary in the degree of difficulty	Individual	Computerized	No	Single-component CCT
Kallio et al. [54]	Cognitive training stimulating sub-skills of executive functioning (selective attention, working memory, cognitive flexibility, and planning)	Individual	Traditional	No	Single-component TCT
Kang et al. [55]	Cognitive training involving various cognitive functions (visuospatial abilities, language, attention, memory, and calculation). Each treatment session focuses on a single function	Individual	Traditional	No	Single-component TCT
Koltai et al. [58]	MCP (involves multiple strategies): space retrieval, face-name recall strategy, verbal elaboration, concentration/over repetition, external aids, and coping strategies	Individual and group	Traditional	No	Single-component TCT
Lee et al. [61]	Cognitive training incorporating errorless learning, spaced retrieval, and vanishing cues. Training components included basic training on various memory types, including sensory memory (visual and auditory), working memory, and prospective memory; memory strategies for using mnemonics, learning principles, and also on name/face association; advanced memory training on the application of strategies to ADL, including home-making, habit training, money management, shopping, and community-living skills	Individual	Traditional	No	Single-component TCT
Loewenstein et al. [64]	Cognitive rehabilitation training focused on face-name association using space retrieval and dual cognitive support, time-and-space orientation using rehearsal and calendars; procedural and motor memory, sustained attention and visuomotor processing, and calculation	Individual	Traditional	No	Single-component TCT
Shyu et al. [81]	Cognitive training based on Baddley and Hitch’s working memory model (phonological loop, visual-spatial sketchpad, and central executive system). The three components were incorporated into the computer games. Progressive difficulty levels were adopted	Individual	Computerized	No	Single-component CCT
Tanaka et al. [88]	Reality orientation, seated physical exercises, cognitive training or cognitive stimulation (music-based activities, reminiscence, facial expression, stimulation of verbal fluency), and cool down	Group	Traditional	Yes: elements of CS, ROT, and physical activity	Multi-component TCT
Trebbastoni et al. [89]	Brief introduction and cognitive training targeting temporal and spatial orientation (i.e., recognizing and recalling the date and the place, using environmental aids), memory, attention, language, visuospatial functions, and executive functions	Group	Traditional	Yes: elements of ROT	Multi-component TCT
Tsantali et al. [90]	Cognitive training targeting semantic memory and naming (i.e., filling the blanks in sentences and paragraphs, semantic categorization, finding synonyms and opposites, categorial fluency, finding similarities and differences) and retrieval (i.e., teaching strategies for improving delayed recall, comprehension, and guided writing)	Individual	Traditional	No	Single-component TCT
Yang and Kwak [99]	Cognitive training targeting seven cognitive domains (attention, spatiotemporal abilities, memory, executive functioning, language, calculation, and auditory perception)	Individual	Computerized	No	Single-component CTC
Cognitive rehabilitation studies					
Reference	Description	Modality	Form	Multi-component	CS type
Amieva et al. [4]	CR focused on the selection of meaningful activities and adaptation of the program to the patient’s abilities	Individual	Traditional	No	Single-component CR
Brueggen et al. [13]	The intervention focused on six modules: (i) identification of problems and definition of treatment goals, (ii) use of external memory aids, (iii) implementation of daily routine activities, (iv) organization of pleasurable and meaningful activities, (v) reminiscence, and (vi) evaluation of achieved goals	Group	Traditional	No	Single-component CR
Clare et al. [26]	The intervention focused on a personally meaningful goal addressing practical aids and strategies, techniques for learning new information (e.g., face-name learning), and techniques for stress management	Individual	Traditional	No	Single-component CR
Clare et al. [25]	The intervention focused on three personal meaningful goals using a problem-solving approach and applying emotional regulation and behavioral activation strategies, reviewing and optimizing strategies used, providing practice in maintaining attention, and signposting to relevant services	Individual	Traditional	No	Single-component CR
Clarkson et al. [28]	Training and support of participants in using a package of customized memory aids	Individual	Traditional	No	Single-component CR
Kim [57]	The intervention focused on personal meaningful goals: practical strategies and aids, compensation strategies, and techniques for stress management. Combined with tasks of CT (time-and-place orientation, matching faces and names, exercises of memory, and sustained attention)	Individual and group	Traditional	Yes: elements of CT	Multi-component CR

*ADL* activities of daily living, *CCT* computerized cognitive training, *CR* cognitive rehabilitation, *CS* cognitive stimulation, *CSP* cognitive stimulation program, *CST* cognitive stimulation therapy, *CT* cognitive training, *MCP* memory and coping program, *ROT* reality orientation therapy, *TCT* traditional cognitive training

### Quality assessment

The quality of the included studies was assessed by a couple of independent reviewers. Interrater agreement was > 90%. The final quality assessment is reported in Supplementary Fig. S2a, Supplementary Fig. S2b, Supplementary Fig. S2c. The overall quality of studies on CS was moderate to high. Overall, 14 out of 30 studies had a low risk of bias [2, 11, 14, 23, 30, 52, 53, 66, 71, 73, 74, 87, 98, 100], 13 had a moderate risk of bias [3, 7, 8, 10, 12, 29, 35],H. J. [56, 68, 78, 79, 86, 90], and three had a high risk of bias [16, 17, 70].

The overall quality of included studies on CT was moderate. Four out of 19 studies had a low risk of bias [4, 9, 15, 20], 10 had a moderate risk of bias [31, 54, 55, 58, 61, 64, 81, 89, 90], and five had a high risk of bias [32, 37, 45, 88, 99].

The overall quality of studies on CR was high. Four studies had a low risk of bias [4, 25, 26, 28], one had a moderate risk of bias [57], and one had a high risk of bias [13].

The main domains for which a high risk of bias was reported were (i) random sequence generation (selection bias), (ii) allocation concealment (selection bias), and (iii) blinding of participants and personnel (performance bias). Many of the included studies either did not report the method of randomization and allocation concealment or reported inadequate methods. Moreover, studies either did not ensure the blinding of participants and staff or reported no information on this domain.

### Demographic and clinical characteristics of the included population

Overall, studies on CS included a total of 2075 PLwD. Of these, 1106 were enrolled in the intervention groups. Participants in the intervention groups had a mean age at baseline ranging from 66.1 to 88.3 years (data not reported in 4 studies) and were for the majority women (66.6%) (data from 24 studies). The mean Mini-Mental State Examination (MMSE) [36] score at baseline ranged from 11.5 to 24.5 (data not available for 4 studies). A total of 969 PLwD were enrolled in the control groups of studies on CS. Participants in the control groups had a mean age at baseline ranging from 68.3 to 86.5 years (data not collected in four studies) and were for the majority women (64.2%) (data from 24 studies). The MMSE at recruitment ranged from 13 to 25.3 (data not available for four studies). The activities performed by the control group were generally limited to usual care or non-specific cognitive tasks.

Studies on CT enrolled a total of 1099 PLwD. Of these, 545 were enrolled in the experimental groups. Participants in the intervention groups had a mean age at baseline ranging from 65.9 to 88.1 years (data not available for two studies) and were for the majority women (data from 14 studies). The mean MMSE score at baseline ranged from a minimum mean score of 15.3 to 25.5 (one study did not provide this data). A total of 554 PLwD were enrolled in the control groups. Participants in the control groups had a mean age at baseline ranging from 66.6 to 84.2 years (data from 16 studies) and were for the majority women (58.7%) (data from 14 studies). The mean MMSE score at baseline ranged from 15.6 to 26.6 (data not considering one study due to lack of full text). The activities performed by the control group were generally limited to usual care or non-specific cognitive tasks.

Studies on CR enrolled a total of 1362 PLwD. Of these, 685 were enrolled in the experimental groups. Participants in the intervention groups had a mean age at baseline ranging from 70.4 to 79.6 years, were for the majority women (52.8%), and had a mean MMSE score at baseline ranging from 21.6 to 23.8. A total of 677 PLwD were enrolled in the control groups. Participants in the control groups had a mean age at baseline ranging from 69.8 to 79.5 years, were for the majority women (53.4%), and had a mean MMSE score at baseline ranging from 21.6 to 24. The activities performed by the control group were generally limited to usual care.

In most of the included studies, global cognitive functioning was evaluated using the MMSE. Functional abilities were assessed using different tools such as the Activities of Daily Living (ADL) [60], the Activities of Daily Living Questionnaire (ADL-Q) [15], and the Bayer-Activities of Daily Living (B-ADL) [48]. The assessment of quality of life (QoL) was performed using the following tools: Quality of Life in Alzheimer’s Disease Scale (QOL-AD) [65], health-related quality of life (HRQoL) [82], and quality-of-life assessment in Dementia (DEM-QOL) [84].

Additional information is reported in Table S1.

### Meta-analysis

#### Group cognitive stimulation

**Fig. 2:**
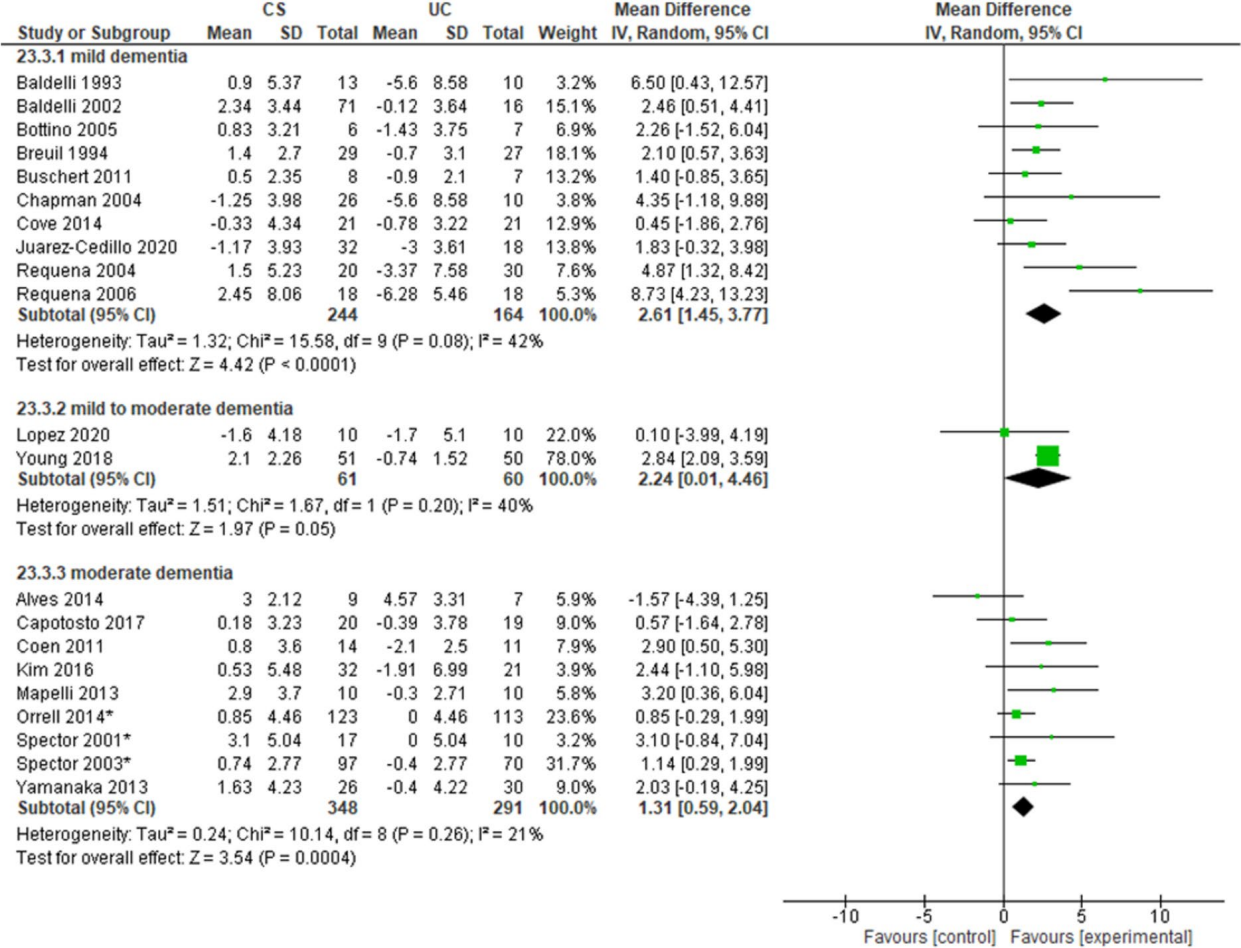
Forest plot for group cognitive stimulation effect on cognitive functions (MMSE). Note. Chi^2^: heterogeneity test; CI: confidence interval; CS: cognitive stimulation; df: degrees of freedom; *I*.^2^: heterogeneity index; IV: inverse variance method; MMSE: Mini-Mental State Examination; Random: random effects model; SD: standard deviation; UC: usual care; *Z*: test for overall mean difference. *Data calculated by the NICE Guideline working group (NG97)

Overall, 24 studies investigated the effectiveness of group sessions of CS in PLwD. When considering cognitive functions, 10 studies [7, 8, 11, 12, 14, 23, 30, 52, 78, 79] reported an improvement in the intervention group compared to the control group in MMSE scores in people with mild dementia (MD = 2.61, 95% CI 1.45–3.77, *n* = 408, *I*^2^ 42%, moderate certainty). Two studies [66, 100] reported an improvement in people with mild-to-moderate dementia (MD = 2.24, 95% CI 0.01–4.46, *n* = 121, *I*^2^ 40%, very low certainty). Nine studies [3, 17, 29],H. J. [56, 68, 74, 86, 87, 98] reported an improvement in people with moderate dementia (MD = 1.31, 95% CI 0.59–2.04, *n* = 639, *I*^2^ 21%, moderate certainty) (Fig. 2). Two studies [2, 66] reported an improvement in the intervention group compared to the control group in cognitive functions measured with ADAS-Cog in people with mild-to-moderate dementia (MD =  − 2.76, 95% CI − 4.7 to − 0.83, *n* = 125, *I*^2^ 0%, moderate certainty). One study [52] reported no differences between groups in the same outcome in people with mild dementia (MD =  − 4.21, 95% CI − 10.26–1.84, *n* = 50, *I*^2^ n.a., low certainty). One study [10] on 57 participants with dementia at an unspecified degree of severity reported no differences between groups in cognitive functions assessed with both ADAS-Cog (MD =  − 5.89, 95% CI − 11.01–0.77, *I*^2^ n.a., very low certainty) and MoCA scale (MD = 3.59, 95% CI 0.72–6.46, *I*^2^ n.a., very low certainty).

When considering functional abilities, four studies [7, 8, 11, 35] reported no differences between groups in people with mild dementia (SMD = 0.19, 95% CI − 0.2–0.57, *n* = 142, *I*^2^ 0%, low certainty). Two studies [17, 74] reported no differences between groups in people with moderate dementia (SMD = 0.07, 95% CI − 0.17–0.31, *n* = 275, *I*^2^ 0%, low certainty) (Supplementary Fig. S3).

Seven studies [3, 17, 29],H. J. [56, 74, 87, 98] reported an improvement in the intervention group compared to the control group in quality of life of people with moderate dementia (SMD = 0.25, 95% CI 0.09–0.41, *n* = 595, *I*^2^ 0%, moderate certainty). Three studies [14, 23, 30] and one study [2] reported no differences between groups respectively in people with mild dementia (SMD = 0.09, 95% CI − 0.29–0.46, *n* = 111, *I*^2^ 0%, low certainty) and mild-to-moderate dementia (MD = 0.47, 95% CI − 1.11–2.05, *n* = 105, *I*^2^ n.a., low certainty) (Supplementary Fig. S4).

#### Individual cognitive stimulation

Overall, six studies investigated the effectiveness of individual sessions of CS.

**Fig. 3:**
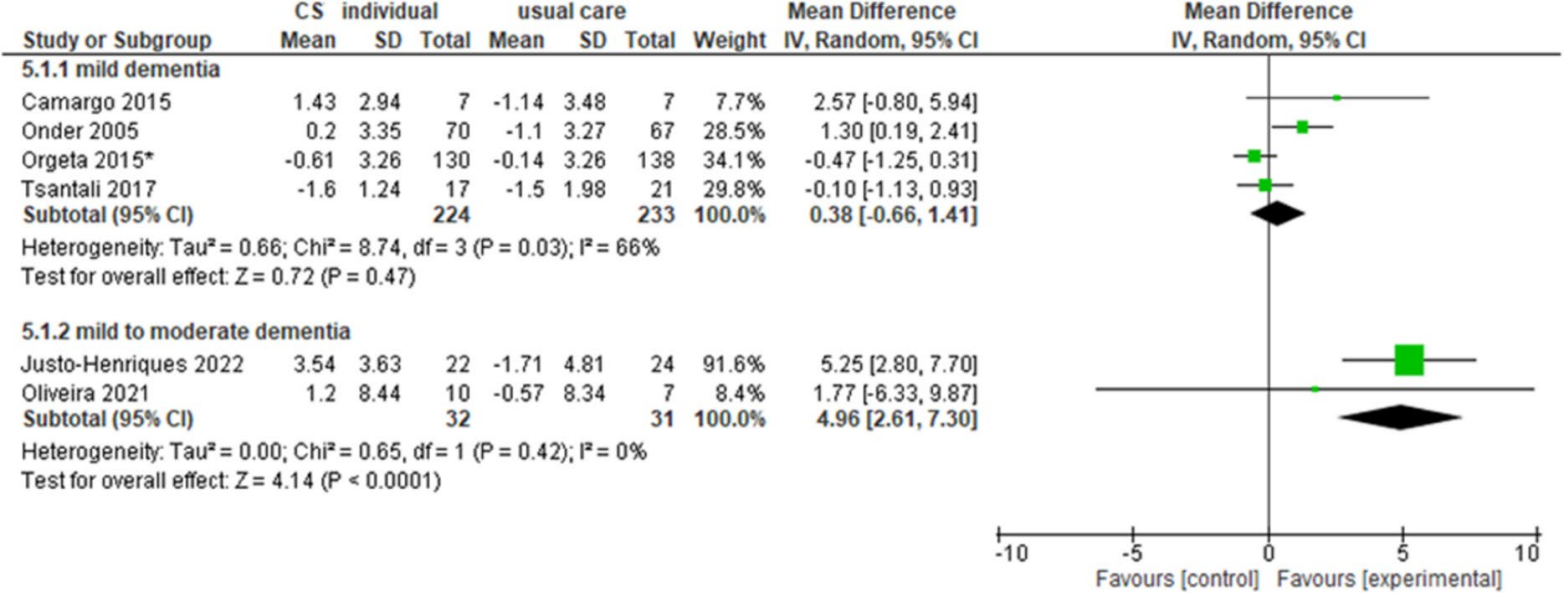
Forest plot for individual cognitive stimulation effect on cognitive functions (MMSE). Note. Chi^2^: heterogeneity test; CI: confidence interval; CS: cognitive stimulation; df: degrees of freedom; *I*.^2^: heterogeneity index; IV: inverse variance method; MMSE: Mini-Mental State Examination; Random: random effects model; SD: standard deviation; *Z*: test for overall mean difference. *Data calculated by the NICE Guideline working group (NG97)

When considering cognitive outcomes, four studies [16, 71, 73, 90] reported no differences between groups in cognitive functions measured with MMSE in people with mild dementia (MD = 0.38, 95% CI − 0.66–1.41, *n* = 457, *I*^2^ 66%, very low certainty) (Fig. 3). Two studies [53, 70] and one study [53] reported an improvement in the intervention group compared to the control group in cognitive functions measured respectively by MMSE (MD = 4.96, 95% CI 2.61–7.3, *n* = 63, *I*^2^ 0%, moderate certainty) (Fig. 3) and MoCA (MD = 7.01, 95% CI 3.91–10.11, *n* = 46, *I*^2^ n.a., moderate certainty) in people with mild-to-moderate dementia. Two studies [71, 73] reported no significant differences between groups in functional abilities in people with mild dementia (SMD = 0.15, 95% CI − 0.04–0.35, *n* = 406, *I*^2^ 0%, moderate certainty).

One study on 272 participants [73] and one study on 46 participants [53] reported no significant differences in the quality of life of people with mild dementia (MD =  − 0.02, 95% CI − 1.04–1, *I*^2^ n.a., moderate certainty) and mild-to-moderate dementia (MD = 4.14, 95% IC − 0.07–8.35, *I*^2^ n.a., low certainty).

#### Group cognitive training

**Fig. 4:**
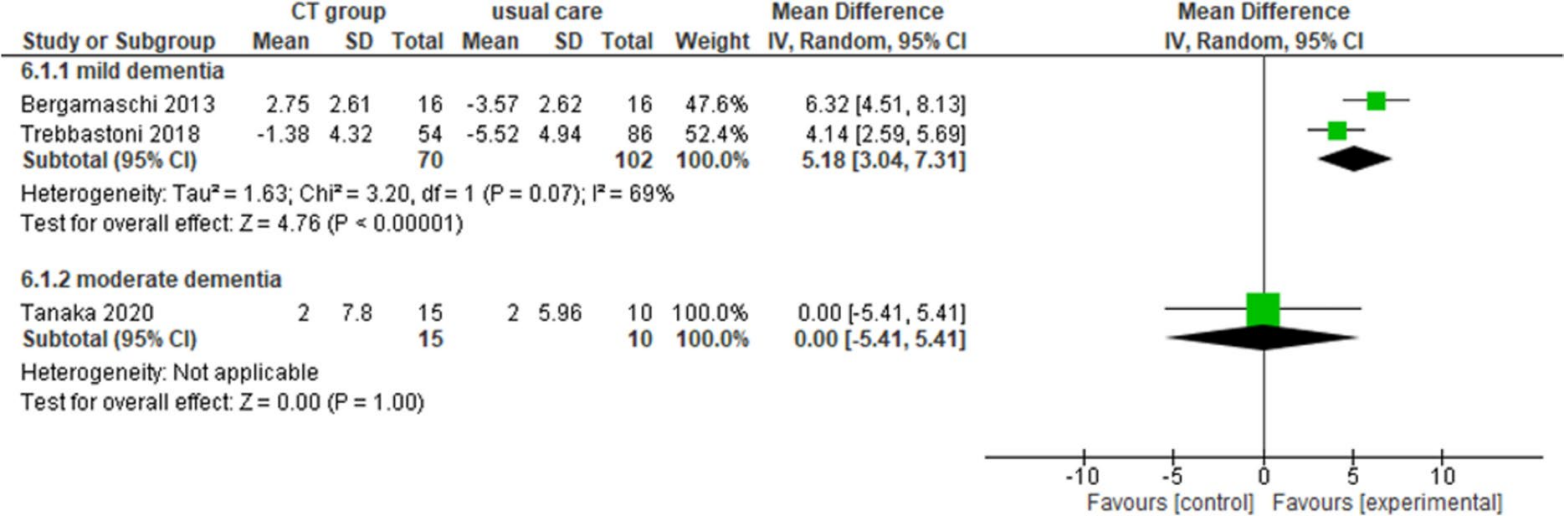
Forest plot for group cognitive training effect on cognitive functions (MMSE). Note. Chi^2^: heterogeneity test; CI: confidence interval; CT: cognitive training; df: degrees of freedom; *I*^2^: heterogeneity index; IV: inverse variance method; MMSE: Mini-Mental State Examination; Random: random effects model; SD: standard deviation; *Z*: test for overall mean difference

Overall, five studies investigated the effectiveness of group sessions of CT. Of the three studies that investigated cognitive outcomes, two studies [9, 89] reported an improvement in the intervention group compared to the control group in cognitive functions in the treated group compared to the control group in people with mild dementia (MMSE: MD = 5.18, 95% CI 3.04–7.31, *n* = 172, *I*^2^ 69%, very low certainty), while one study [88] reported no differences between groups in the same outcome in people with moderate dementia (MMSE: MD = 0.00, 95% CI − 5.41–5.41, *n* = 25, *I*^2^ n.a., very low certainty) (Fig. 4). The same study [88] also showed no differences in quality of life (MD = 3.4, 95% CI − 1.32–8.12, *I*^2^ n.a., very low certainty).

Finally, three studies [4, 9, 15] with mild dementia reported no differences between groups in daily activities assessed with ADL (SMD = 0.13, 95% CI − 0.34–0.60, *n* = 299, *I*^2^ 57%, very low certainty) (Supplementary Fig. S5).

#### Individual cognitive training

**Fig. 5:**
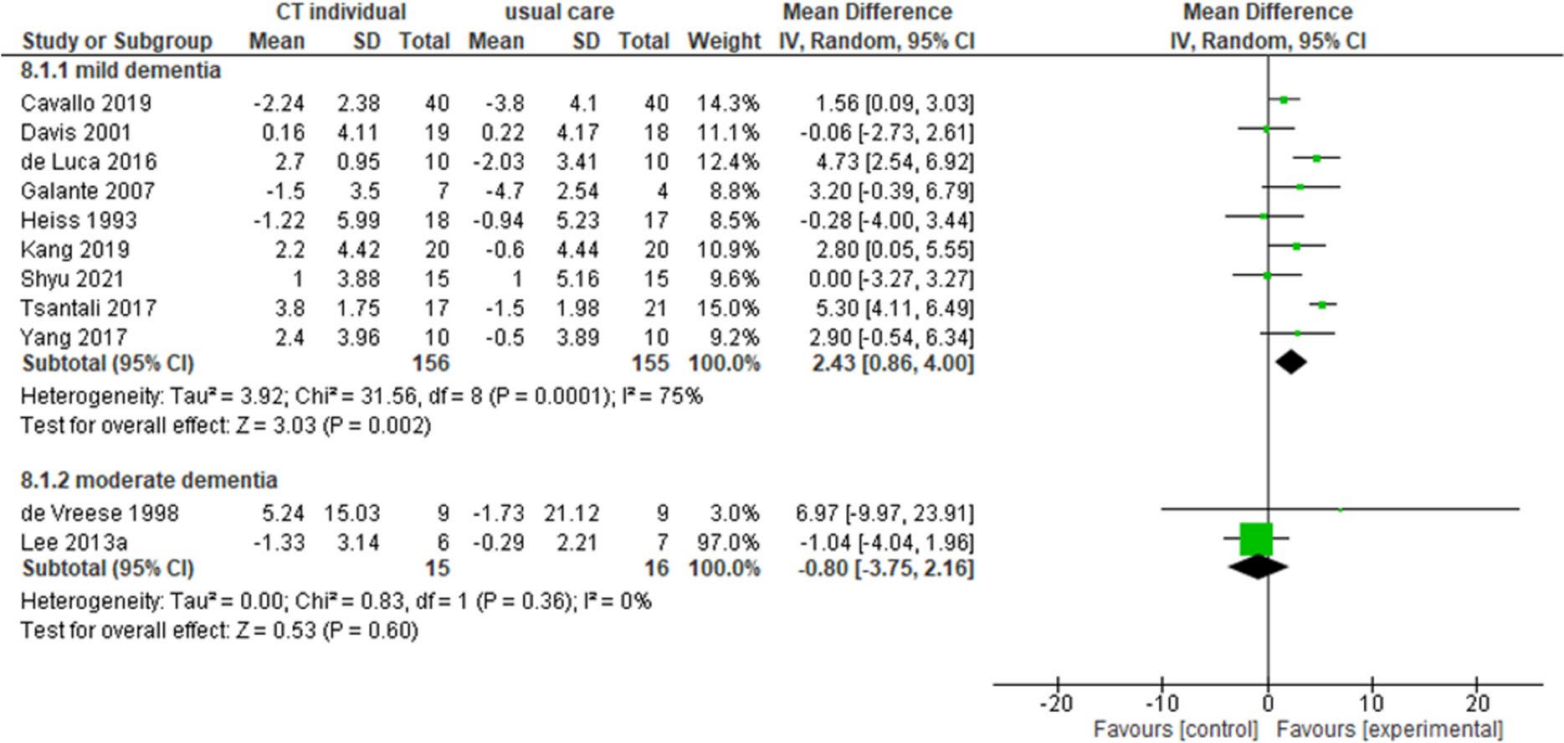
Forest plot for individual cognitive training effect on cognitive functions (MMSE). Note. Chi^2^: heterogeneity test; CI: confidence interval; CT: cognitive training; df: degrees of freedom; *I*^2^: heterogeneity index; IV: inverse variance method; MMSE: Mini-Mental State Examination; Random: random effects model; SD: standard deviation; *Z*: test for overall mean difference

Overall, 14 studies investigated the effectiveness of individual sessions of CT in PLwD. Nine studies [20, 31, 32, 37, 45, 55, 81, 90, 99] reported an improvement in the intervention group compared to the control group in cognitive functions measured with MMSE in people with mild dementia (MD = 2.43, 95% CI 0.86–4.00, *n* = 311, *I*^2^ 75%, very low certainty). One study on 147 participants with mild-to-moderate dementia [54] and two studies on 31 participants with moderate dementia [33, 61] reported no differences between groups in the same outcome measured respectively with MMSE (MD =  − 0.80, 95% CI − 3.75–2.16, *I*^2^ 0%, very low certainty) (Fig. 5) and ADAS-Cog (MD =  − 0.90, 95% CI − 2.36–0.56, *I*^2^ n.a., low certainty).

When considering functional abilities, two studies on 277 participants [37, 64] and two studies on 31 participants [33, 61] reported no differences between groups in ADL respectively in people with mild dementia (SMD = 0.02, 95% CI − 0.22–0.25, *I*^2^ 0%, very low certainty) and moderate dementia (SMD = 0.42, 95% CI − 0.29–1.14, *I*^2^ 0%, low certainty) (Supplementary Fig. S6). One study on participants with mild-to-moderate dementia [54] reported no differences between groups in health-related quality of life (MD = 0.00, 95% CI − 0.03–0.03, *n* = 147, *I*^2^ n.a., low certainty).

#### Cognitive rehabilitation

Overall, six studies investigated the effectiveness of individual sessions of CR in people with mild dementia. When considering cognitive outcomes, one study on 16 participants [13] reported no differences between groups in cognitive functions measured with MMSE (MD = 0.87, 95% CI − 0.96–2.70, *I*^2^ n.a., low certainty).

When considering functional abilities, four studies [4, 25, 26],S. [57] reported an improvement in the intervention group compared to the control group in this outcome (SMD = 0.52, 95% CI 0.04–1.00, *n* = 728, *I*^2^ 86%, low certainty) (Supplementary Fig. S7). Two studies [13, 28] reported no differences between groups in ADL measured with different tools (SMD =  − 0.15, 95% CI − 0.89–0.59, *n* = 484, *I*^2^ 59%, very low certainty) (Supplementary Fig. S8). Five studies [4, 13, 25, 26],S. [57] reported no differences between groups in quality of life of people with mild dementia measured with different tools (SMD = 0.22, 95% CI − 0.08–0.53, *n* = 789, *I*^2^ 62%, very low certainty) (Supplementary Fig. S9).

### Narrative summary

#### Cognitive stimulation therapy, reality orientation therapy, and other CS protocols

Many CS interventions were based on the CST protocol. One study [30] investigated group sessions of CST in people with mild dementia, reporting no differences between groups in MMSE scores (MD = 0.45, *p* > 0.05). However, two studies [2, 100] investigated group sessions of CST in people with mild-to-moderate dementia and reported a significant effect of the intervention on global cognition measured with MMSE (MD = 2.84, *p* < 0.01) and Adas-Cog (MD =  − 2.86, *p* < 0.05), respectively. Six studies [17, 29, 74, 86, 87, 98] investigated the effectiveness of group sessions of CST in people with moderate dementia and reported an improvement in the intervention group compared to the control group in cognitive functions, measured with MMSE. The highest improvements in MMSE scores were reported by two studies [29, 87], with one study reporting an MD of 2.90 (95% CI 0.50–5.30) [29] and one study reporting an MD of 1.14 (95% CI 0.29–1.99) [87] points at follow-up. The highest reported MD was 3.10 [86], but it was not statistically significant (*p* = 0.08). One study [10] investigated group sessions of virtual CST and reported no significant differences between groups in MoCA and Adas-Cog scores.

Two studies [53, 73] investigated individual sessions of CST. One study [53] on people with mild-to-moderate dementia reported an improvement in the intervention group compared to the control group in MMSE scores (MD = 5.25, *p* = 0.000). However, the other study [73] reported no significant differences between groups (MD =  − 0.47, *p* = 0.23) in cognitive functions in patients with mild dementia. Overall, no differences between groups were reported for both group and individual CST in functional abilities measured with ADL, but five studies [17, 29, 74, 87, 98] reported an improvement in quality of life of people with moderate dementia treated with group sessions of CST.

Five studies [7, 8, 16, 35, 71] investigated the effectiveness of interventions based on ROT in people with mild dementia. Two studies [7, 8] investigated group sessions of ROT, reporting an improvement in the intervention group in cognitive functions (MMSE) with one study reporting an MD of 6.50 (95% CI 0.43–12.57) [8] and one study reporting an MD of 2.46 (95% CI 0.51–4.41) [7]. One study [71] reported a positive effect on cognitive functions in people with mild dementia treated with individual sessions of ROT (MMSE: MD = 1.30, *p* = 0.02). One study [16] reported no differences in cognitive functions between individual sessions of ROT and usual care (MD = 2.57, *p* = 0.14). No studies investigated the effectiveness of ROT in improving quality of life, and only two studies [7, 8] investigated its effect on functional abilities, reporting no significant differences between groups.

A total of 13 studies [3, 11, 12, 14, 23, 52],H. J. [56, 66, 68, 70, 78, 79, 90] investigated the effectiveness of either single-component or multi-component CSP. Most of these studies investigated the effectiveness of CSP in people with mild dementia, reporting heterogeneous results. Three studies [12, 78, 79] reported an improvement in MMSE scores in people treated with group sessions of single-component CSP. However, four studies [11, 14, 23, 52] reported no differences in patients with mild dementia. Three studies [3],H. J. [56, 68] investigated the effectiveness of single-component CSP in people with moderate dementia, with only one study [68] reporting an improvement in MMSE scores in people with moderate dementia (MD = 3.20, *p* < 0.001). Only one study [56] investigated the effectiveness of group sessions of multi-component CSP in people with moderate dementia, reporting no significant differences in MMSE scores. No differences between groups were also reported for both single-component and multi-component CSPs in functional abilities and quality of life.

#### Computerized cognitive training (CCT) vs. traditional cognitive training (TCT)

Studies investigating group sessions of CT [4, 9, 15, 88, 89] usually administered the TCT. Overall, these studies reported a significant improvement in cognitive functions measured with MMSE in people with mild dementia.

A higher heterogeneity was found among studies investigating individual sessions of CT. A total of six studies [20, 32, 37, 45, 81, 99] investigated CCT in people with mild dementia, reporting an overall improvement in the intervention group in cognitive functions. However, only two studies [20, 32] reported a significant improvement in MMSE scores, with one study reporting an MD of 1.56 (95% CI 0.09–3.03) [20] and one study reporting an MD of 4.73 (95% CI 2.54–6.92) [32]. No significant differences between groups were reported in the ADL scores. Seven studies [31, 33, 54, 55, 58, 64, 90] investigated the effectiveness of TCT. Only two studies [55, 90] reported a significant improvement in the intervention group compared to the control group in cognitive functions measured with MMSE in people with mild dementia, with one study reporting an MD of 2.80 (95% CI 0.05–5.55) [10, 55] and one study reporting an MD of 5.30 (95% CI 4.11–6.49) [90]. Two studies [33, 64] investigated the effect of TCT on functional abilities in people with mild and moderate dementia, reporting no differences between groups in ADL scores.

#### Caregivers

Four out of the 30 studies on CS investigated the effects of this intervention on caregiver-related outcomes [3, 10, 11, 71]. One study [10] reported a significant difference between groups in caregiver burden measured with the Zarit Burden Inventory (ZBI) [101], with a significant decrease in ZBI scores in the intervention group (MD = 3.78, *p* = 0.001) compared to a significant increase in ZBI scores in the control group (MD = 2.06, *p* = 0.002). However, three studies [3, 11, 71] reported no differences between groups in anxiety, depression, burden, and general health measured using different questionnaires.

Only one study [4] investigated the effect of CT on caregiver-related outcomes and reported no differences between groups in ZBI scores at 3- and 24-month post-intervention.

Five out of the six studies on CR evaluated investigated its effects on different caregiver outcomes [4, 13] [25, 26, 28]). Overall, the studies suggested a positive effect of CR. One study [4] reported an improvement at 3 months in the intervention group compared to usual care in caregivers’ burden measured with ZBI (*p* = 0.01). This result remained significant at 24 months post-intervention (*p* = 0.05). One study [26] reported an improvement in the intervention group in social relationships measured with the social relationship subscale of the World Health Organisation Quality of Life Instrument—brief version (WHOQOL-BREF, Whoqol Group, [44]) at 8 weeks and 3 months, respectively.

## Discussion

This review analyzed available evidence on the effectiveness of cognitive stimulation (CS), cognitive training (CT), and cognitive rehabilitation (CR) on cognitive function, activities of daily living, and quality of life in people living with dementia (PLwD) and their effect on caregivers’ burden and mental health. The effectiveness of COTs has been already investigated through systematic reviews and meta-analyses [1, 94, 97]. However, to our knowledge, this is the first study evaluating the effectiveness of CS, CT, and CR taking into account the modality (individual or group session), system for delivering the exercises, and form of the intervention.

Results suggested that CS appears to be an overall effective and cost-effective intervention, with improvements observed in people at any stage of dementia and in all considered outcomes, and the high quality of the involved studies further supports this evidence.

When considering cost-effectiveness, data were insufficient to support any clear indications of the sustainability of these interventions. As reported in previous studies specifically focusing on the impact of these interventions on the use of resources, further health economic data are needed (Livingstone et al., [63], [34]). Moreover, as reported in the analyses performed by the Italian guideline (ISS, [50]), the specific costs can widely vary depending on the context where the intervention is meant to be implemented. Therefore, considering the relevance and overall effectiveness of these interventions in the management of PLwD and their having the stronger evidence of cost-effectiveness [34], cost analyses accounting for the specific context where each intervention is meant to be implemented (e.g., Health technology assessments) could support decision makers in assessing the financial impact of implementing health services or interventions.

When considering the clinical effectiveness of these interventions, both individual and group sessions of CS were reported to be effective in improving global cognitive functions. Specifically, individual CS was reported to improve cognitive functions in people with mild-to-moderate dementia, while group CS was reported as effective in people with mild, mild-to-moderate, and moderate dementia. However, these results were limited by a moderate to high heterogeneity. This may be due to differences in the size and characteristics of the considered samples, which may cause differences in the response to the intervention. This heterogeneity affects the generalizability of results. Furthermore, group CS was reported to improve QoL in people with moderate dementia, and this result could be explained by the social component of CS. Moreover, research explored which factors could enhance the effectiveness of this intervention [94]. Three elements were proposed as potentially being the main key factors when integrated into these interventions: cognitive exercises with a broad spectrum, social interaction, and support, with all of them being based on a person-centered approach safeguarding and ensuring the dignity of people with dementia [93]. Recent qualitative studies highlighted the importance of these elements in both group and individual CS [40, 72].

When considering CT, both individual and group interventions were reported to improve global cognitive functions in people with mild dementia. These results are strongly consistent with the intrinsic nature of this type of intervention. CT was reported to be more effective in people with mild dementia than in people with severe dementia. This could be due to CT being based on more cognitively demanding activities compared to less standardized and more ecologically (namely, closer to everyday tasks) focused task-based interventions like CS. Another possible reason could be the difficulty in applying domain-specific and standardized learning to daily tasks and non-domain-specific assessments, especially in advanced dementia stages [77]. As suggested by Owen et al. [75], while improvements may be observed on tasks trained with cognitive training, transfer is seldom observed, and if it does occur, it typically involves tasks related to the same trained cognitive function [75]. However, the certainty of the evidence for CT was lower compared to evidence on CS and limited by high heterogeneity, which could be due to differences in the characteristics and type, length, and intensity of the interventions, and this should be taken into account when considering results from these studies.

Finally, CR was found to be effective only on functional abilities. This is consistent with the purpose of CR, that is, to preserve or improve functioning in everyday life [13],NG97, [69]). However, the included studies did not consistently report an impact of CR on functional abilities, and results from the meta-analysis highlighted a high heterogeneity. This heterogeneity may be due to the different sensitivity of the assessment tools used across studies [13], to differences in the size and characteristics of the enrolled sample, as for the other interventions, and to differences in the implementation of the intervention in terms of activity, length, and intensity. Moreover, included studies only enrolled people with mild dementia. This research focus can be considered reasonable, as CR interventions require specific cognitive functions (e.g., memory, planning, and abstraction ability) to be preserved to be effectively implemented and applied in daily life [13]. Further limitations were the smaller number of available studies and the low frequency and intensity with which intervention was carried out. The quality assessment of the studies mostly showed a moderate risk of bias.

A common limitation to all three types of interventions was the variability in the length and intensity of treatments, with studies often implementing short-term interventions with very low intensity. This may potentially explain both the heterogeneity observed within each type of intervention and the lack of efficacy reported by some trials. Notably, intensity and extensiveness seem to be key elements for the effectiveness of cognitive interventions, providing more resilience to neuropathology and increasing cognitive and brain reserve [62]. One further issue was related to the broader scope of the interventions considered. As underlined by both the NG97 (NG97, [69]) and the Italian guideline (ISS, [50]), despite literature homogeneously reporting the basic principles of each intervention, included studies showed considerable variability in how the interventions were performed. Specifically, some studies implemented different interventions combining or supplementing them with other non-pharmacological interventions. Some studies on CS, for example, implemented CST protocol, while others included different CSPs that were not based on a specific protocol. Results showed that group CST was effective in enhancing cognitive abilities, especially in people with mild-to-moderate and moderate dementia. Individual CST, although applied in fewer studies, also appeared to have a positive effect in people with mild-to-moderate dementia. Some of the studies on both group and individual CS adopted a ROT protocol in mild dementia, reporting efficacy on cognitive abilities for both group and individual intervention. This intervention plays a crucial role as it is one of the first COTs investigated in people with dementia and because its core principles are incorporated in CST and many interventions that are part of CSPs [18, 86, 87]. A lower heterogeneity in CR interventions was observed, which could be also due to the lower number of included studies. However, the structure of this intervention itself allows for heterogeneity, as it needs to be highly personalized. CR interventions are reported to be more effective the more they are personalized and targeted to goals relevant to the person [13]. Studies on CT, on the other hand, reported a high variability in how the intervention was implemented. The targeted functions varied considerably across studies. In recent years, partly due to the introduction of CCT, the importance of personalizing the intervention has been increasingly emphasized [6], which is easier to pursue in clinical practice than in research. The included studies were widely heterogeneous in terms of length and intensity of interventions and in terms of which cognitive functions were specifically trained. Evidence suggested that CT seems to be effective both in its traditional (TCT) form and in its computerized (CCT) form. This is particularly relevant for those situations where face-to-face therapy is not feasible for all PLwD. Offering the opportunity to tailor and personalize the intervention, especially through CCT, could allow to maximize its potential benefits. Several different factors, such as the underlying cause of dementia, individual characteristics (e.g. cognitive reserve), and sociodemographic characteristics (e.g., educational level, cultural factors) can contribute to the variability in the response to treatment. The potential influence of cognitive resources on treatment response is a still widely debated issue. According to the magnification view, individual differences in gains from cognitive training could be due to baseline differences in cognitive resources [67]. This would mean that group differences should be magnified after treatment, gains should be positively correlated with baseline performance, and interindividual differences should be higher after treatment compared to baseline. In contrast, according to the compensation view, people who already have good assets have a lower probability of showing improvement after treatment as they are already functioning at an optimal level [67]. On this basis, this view assumes that gains should be negatively correlated with baseline performance and that interindividual differences should be lower after treatment [67]. Results from the meta-analysis on CT interventions seem to be consistent with the first hypothesis. That is even more plausible when considering the magnification hypothesis along with the concepts of cerebral and cognitive reserve. These two types of reserve allow to compensate for deficits due to neurocognitive disorders by providing a buffer at the biological and alternative cognitive strategies level. This suggests that people in earlier stages of dementia could benefit more from this intervention as they have more residual cerebral and cognitive resources. CT may therefore be less effective in people with more severe dementia due to the progression of neurodegeneration. Accordingly, the Italian guideline reported that CT is effective in people with MCI in a broader way compared to people with dementia. However, to our knowledge, few studies investigated the correlation between cerebral and cognitive reserve and the efficacy of COTs in people with dementia. However, cognitive reserve and therefore the effectiveness of COTs are linked to several more complex factors involving lifestyle, cultural factors, and overall physical and emotional well-being [24]. Lifestyle habits, including physical exercise, diet, sleep patterns, alcohol consumption, smoking, and use of medications for previous or current medical conditions, have been reported to have an impact on the general health status and cognitive performances, thus potentially affecting the overall dimension of the effect of COTs. However, none of the studies included in this systematic review and meta-analysis specifically investigated the role of these aspects in relation to the outcomes. Moreover, cultural factors can limit the access and effectiveness of COTs. Culturally adapting COTs that have been proven to be effective and maintaining their core components can make them acceptable and feasible in a wider population, reducing inequalities in accessing these interventions [51]. The effectiveness of COTs also appears to be deeply interconnected with the unique impact of dementia in the whole life of every single person,therefore, these interventions should also consider and integrate strategies to target the impact of the disease on the person’s sense of self, their motivation, and potential problems affecting their ability to adhere to and accept this type of engaging interventions [24]. The overall respect for PLwD when considering their participation in interventions and the adequate assessment of their ability to consent to participation and comprehend the information they are provided are also part of the main ethical requirements for experimental studies on PLwD [22, 43]. Involving PLwD in trials, in fact, raises unique specific ethical challenges that should be adequately managed to avoid exploiting PLwD while at the same time ensuring their representativeness in research studies. Accurately identifying and discussing these ethical challenges and raising awareness of the complexity of addressing ethical issues in trials on PLwD could help increase the quality of research and therefore the quality of care [43].

A further limitation of analyzed evidence was the heterogeneity in the characteristics of the considered intervention, including the type, length, and intensity. This is also due to the need to tailor and personalize interventions to the individual needs and goals. The need to personalize interventions leads to specificities that hinder the cumulative analysis of evidence. However, tailoring interventions based on the goals, expectations, and need of PLwD and their caregivers is essential to effectively manage their care. The whole process of personalizing interventions should be based on a thorough analysis of the main factors that motivate each person. Discussing personalization without considering this phase risks leading to interventions where the goal is merely to complete each planned task. Personalized activities based on elements that are significant and thus motivating for the person can significantly increase the perceived experiential quality. This type of experience aims at integrating impaired and non-impaired functions and abilities within the human system and stimulating and reactivating problem-solving skills in specific situations and moments. The goals achieved through exercises and tasks that are not based on more complex personalized activities risk being only partially useful and not maintained in the long term as the person may be unable to transfer them in their daily life. The adoption of personalized and meaningful activities, adjusting their intensity and difficulty, should therefore be encouraged within scientific studies based on an interdisciplinary perspective.

Thoroughly exploring these features could have significant implications in understanding the mechanisms underlying the efficacy of COTs in people with dementia, allowing to optimize the interventions and improve their effectiveness.

Dementia is also considered a “social disease” as it affects both people themselves and the social network in which the PLwD is embedded. Therefore, the role of this network, and especially caregivers, becomes crucial in every stage of the disease. The progressive loss of functioning and the potential onset of behavioral and psychological symptoms of dementia (BPSD) can increase the burden on caregivers. Caregivers’ burden refers to the physical, psychological, social, emotional, and economic strain experienced by caregivers of people with a chronic illness [80]. Indeed, a survey conducted by Censis and the Italian Alzheimer’s Disease Association in 2016 found that caregivers of PLwD often experience fatigue and depression, which can lead them to start taking medications due to the impact that caregiving has on their health, with variable percentages depending on the reported symptomatology (e.g., depression, anxiety, sleep disorders) [21]. The Italian guideline reported that the combination of psychoeducational and skill training interventions and cognitive-behavioral therapy can be effective in caregivers and PLwD. Despite COTs not being designed to reduce caregiver burden, we explored whether these interventions can indirectly improve caregiver’s well-being. Only four studies on CS investigated this outcome, reporting no significant results. Studies on CR studies reported a potentially positive effect of the type of interventions on caregivers’ burden. This can be expected as CR intervention aims are set along with the PLwD and often with their caregivers, who are also involved in the management of the intervention. As mentioned, the purpose of CR is to improve or maintain functioning and independence in daily living. Considering how these interventions were reported to be effective in improving functional abilities in PLwD and how involving caregivers in delivering the intervention is often considered as a part of the intervention process, these two factors can be considered as contributing to the observed improvement in caregivers’ well-being.

## Future directions

Further studies should be carried out adopting a more rigorous methodology (e.g., randomization strategies) to investigate the effectiveness of COTs, especially CT and CR interventions, and attempting to address the existing heterogeneity, including standardizing interventions, and reporting detailed data on treatment protocols and on the frequency and intensity of interventions. Few RCTs were available investigating the effectiveness of CR in PLwD. Further studies should be carried out investigating this type of treatment but increasing its frequency, as it is currently not extensively applied (leaving much of its implementation to be carried out by family members thus in an unsupervised manner). Several studies investigated the effectiveness of CT in PLwD. However, their quality was moderate, the frequency and length of interventions were extremely variable, and the ecological validity of the intervention was low. Therefore, future research should focus on reducing risks of bias, implementing CT in a more intensive and extensive form, and including more ecological tasks to facilitate the generalization of its effects.

As mentioned, exploring the potential association between cognitive and cerebral reserve and the effectiveness of COTs, especially CT intervention, could be extremely useful both in clinical practice and to increase knowledge on the mechanisms underlying the effect of these interventions. Exploring the potential role of lifestyle factors and the active involvement of caregivers in delivering interventions could also help design more personalized interventions targeted to the goals and needs of both PLwD and their caregivers. Future research should also explore the impact of these interventions on the well-being of caregivers, who play a crucial role throughout the course of the disease and could often experience significant health consequences from caregiving.

## Supplementary Information

Below is the link to the electronic supplementary material.

Supplementary file1 (PDF 407 KB)

Supplementary file2 (DOCX 1076 KB)

Supplementary file3 (DOCX 107 KB)

Supplementary file4 (DOCX 31 KB)

## Data Availability

The data used in this manuscript are publicly available to the research community in the Italian guideline 'Diagnosis and Treatment of Dementia and Mild Cognitive Impairment', which can be found on the website of the Italian National Guidelines System (SNLG): https://www.iss.it/-/snlg-diagnosi-e-trattamento-delle-demenze.
