# Correction to: The efficacy of cognitive stimulation, cognitive training, and cognitive rehabilitation for people living with dementia: a systematic review and meta‑analysis

**DOI:** 10.1007/s11357-025-01551-7

**Published:** 2025-02-06

**Authors:** Alice Paggetti, Ylenia Druda, Francesco Sciancalepore, Francesco Della Gatta, Antonio Ancidoni, Nicoletta Locuratolo, Paola Piscopo, Luca Vignatelli, Luciano Sagliocca, Antonio Guaita, Piero Secreto, Andrea Stracciari, Paolo Caffarra, Nicola Vanacore, Elisa Fabrizi, Eleonora Lacorte, the Italian Dementia Guideline Working Group

**Affiliations:** 1 National Centre for Disease Prevention and Health Promotion, Italian National Institute of Health, Rome, Italy; 2 Department of Medical and Surgical Sciences, University of Bologna, Bologna, Italy; 3 Department of Human Neuroscience, Sapienza University of Rome, Rome, Italy; 4 Department of Neuroscience, Mental Health and Sense Organs (NESMOS), Faculty of Medicine and Psychology, Sapienza University of Rome, Rome, Italy; 5 Department of Neuroscience, Italian National Institute of Health, Rome, Italy; 6 IRCCS Istituto Delle Scienze Neurologiche Di Bologna, Bologna, Italy; 7 Local Health Unit, Salerno, Italy; 8 Golgi Cenci Foundation, Abbiategrasso, Milan Italy; 9 Alzheimer Unit, Fatebenefratelli Hospital, San Maurizio Canavese, (TO) Italy; 10 Cognitive Disorder Center, Neurology Unit, S.Orsola-Malpighi University Hospital, Bologna, Italy; 11 Dementia Unit AOU, Parma, Italy

**Correction to: GeroScience**

10.1007/s11357-024-01400-z

In this article the author’s name Paolo Caffarra was incorrectly written as Paola Caffarra.

The author group information has been updated.

