# Supplementary material for: The efficacy of cognitive stimulation, cognitive training, and cognitive rehabilitation for people living with dementia: a systematic review and meta-analysis
Source: GeroScience. 2024 Nov 1;47(1):409–44. doi: 10.1007/s11357-024-01400-z (PMC11872969; doi:10.1007/s11357-024-01400-z)
Supplement: Supplementary file 1 — Supplementary file1 (PDF 407 KB) [file 11357_2024_1400_MOESM1_ESM.pdf]

Document S1 Search strategy

Cochrane Library

#1 MeSH descriptor: [Dementia] explode all trees
#2 (dementi* OR pseudodementia):ti,ab
#3 (alzheimer* OR alzeimer* OR (cortical NEAR/4 sclerosis)):ti,ab
#4 ((encephalopath* OR cogniti* OR neurocogniti*) NEAR/4 (aids OR hiv)):ti,ab
#5 ((aphasi* NEAR/4 (primary OR progress*)) OR mesulam* OR ppa OR (ftd NEAR/4 temporal)):ti,ab
#6 (((creutzfeldt OR jakob*) NEAR/4 (disease OR syndrome)) OR cjd OR (spongiform NEAR/4 encephalopath*) OR "corticostriatospinal degeneration" OR (pseudosclerosis NEAR/4 spastic)):ti,ab
#7 (binswanger* OR ((subcortic* OR arterisclerotic) NEAR/4 (encephalopath* OR leukoencephalopath*)) OR cadasil*):ti,ab
#8 ((sub NEXT cortic*) NEAR/4 (encephalopath* OR leukoencephalopath*)):ti,ab
#9 ((kosaka NEAR/2 shibayama) OR (neurofibrillary NEXT tangle*) OR dntc):ti,ab
#10 (((frontotemporal OR (fronto NEXT temporal) OR (corticobasal OR (cortico NEXT basal) OR (frontal NEXT lobe))) NEAR/4 (degenerati* or dysfunction)) OR ftld OR ftlds OR ftd OR ftds):ti,ab
#11 (pick* NEAR/1 (complex OR disease* OR syndrome)):ti,ab
#12 ((wilhemsen NEAR/1 lynch) OR ddpac OR (lob* NEAR/4 atroph*)):ti,ab
#13 (huntington* OR ((progressive OR major OR juvenile OR hereditary) NEAR/4 chorea)):ti,ab
#14 (((kluver OR kluever) NEAR/4 bucy) OR (("temporal lobectomy" NEAR/4 behavi*) or ("temporal lobe" NEAR/4 dysfunction))):ti,ab
#15 ((lewy NEXT bod*) OR dlb OR lbd OR dlbd):ti,ab
#16 ((senile NEXT confusion) OR (senile NEXT psychosis) OR senilit*):ti,ab
#17 MeSH descriptor: [Tauopathies] this term only
#18 tauopath*:ti,ab
#19 cerad:ti,ab
#20 Posterior NEXT cortic* NEXT atroph*
#21 sivd:ti,ab
#22 {OR #1-#21}
#23 ((Non-pharma* OR ("non" NEXT pharma*)) NEAR/2 (intervent* OR therap* OR treat* OR stimulat* OR aid* OR techni* OR program* OR care)):ti,ab
#24 MeSH descriptor: [Sensory Art Therapies] explode all trees
#25 MeSH descriptor: [Complementary Therapies] this term only
#26 MeSH descriptor: [Counseling] this term only
#27 MeSH descriptor: [Psychotherapy] this term only
#28 MeSH descriptor: [Aromatherapy] this term only
#29 MeSH descriptor: [Art Therapy] explode all trees
#30 MeSH descriptor: [Color Therapy] this term only
#31 MeSH descriptor: [Dance Therapy] this term only
#32 MeSH descriptor: [Imagery, Psychotherapy] this term only
#33 MeSH descriptor: [Music Therapy] this term only
#34 MeSH descriptor: [Narrative Therapy] this term only
#35 MeSH descriptor: [Play Therapy] explode all trees
#36 ((Sensor* OR multisensor* OR complement* OR alternat* OR music* OR sound* OR acoustic* OR audi* OR dance* OR dancing OR play* OR art* OR touch* OR literat* OR read* OR narrative* OR visual* OR vision* OR light* OR colour* OR color* OR environment* OR massage* OR aroma* OR dawn* OR sunshine OR sunlight OR counsel*) NEAR/1 (intervent* OR therap* OR stimulat* OR aid* OR techni* OR train* OR treat* OR counsel* OR program*)):ti,ab
#37 (snoezelen* OR (senso* NEXT environment*) OR (senso* NEXT room*) OR MSE):ti,ab

#38 (Psychotherap* or psycho-therap* or psychoeducat* or psycho-educat*):ti,ab
#39 (Aromatherap* or chromatotherap*):ti,ab
#40 MeSH descriptor: [Massage] this term only
#41 MeSH descriptor: [Oils, Volatile] this term only
#42 ((Essential or volatile or fragrant) NEAR/1 oil*):ti,ab
#43 MeSH descriptor: [Cognitive Behavioral Therapy] explode all trees
#44 MeSH descriptor: [Behavior Therapy] this term only
#45 ((Cognitive* OR cognition* OR behaviour* OR behavior* OR individual*) NEAR/2 (intervent* OR therap* OR stimulat* OR aid* OR techni* OR train* OR skill* OR rehab* OR treat* OR counsel*)):ti,ab
#46 CBT:ti,ab
#47 MeSH descriptor: [Holistic Health] this term only
#48 ((Validat* or holistic*) NEAR/1 (therap* or method* or techni* or treat* or train* or intervent* or program* or care)):ti,ab
#49 (Memory NEAR/2 (function* or rehab* or therap* or aid* or group* or train* or retraining or support* or stimulation or strateg* or management or managing)):ti,ab
#50 (Reality NEAR/2 orientation):ti,ab
#51 Reminiscence*:ti,ab
#52 MeSH descriptor: [Occupational Therapy] explode all trees
#53 ((Occupation* or Occ) NEAR/2 (therap* or treat* or care* or medicine*)):ti,ab
#54 OT:ti or ergotherap*:ti,ab
#55 MeSH descriptor: [Language Therapy] this term only
#56 MeSH descriptor: [Speech Therapy] this term only
#57 ((Speech or talk* or language*) NEAR/2 (intervent* or therap* or stimulat* or aid* or techni* or train* or treat* or counsel* or method* or program*)):ti,ab
#58 MeSH descriptor: [Activities of Daily Living] this term only
#59 ((Daily NEAR/2 (life or living) NEAR/2 activi*)):ti,ab
#60 ADL:ti
#61 MeSH descriptor: [Exercise Therapy] explode all trees
#62 ((Exercis* or kinesiotherap* or kinesiolo* or physical* or sport*) NEAR/1 (therap* or treat* or intervent* or medicin* or educat*)):ti,ab
#63 ((walk* or swim* or jog* or cycling or bicycl* or treadmill* or gym*) NEAR/1 (therap* or treat* or intervent* or medicin* or educat*)):ti,ab
#64 ((Aerobic OR yoga OR pilates OR "tai chi" OR tai-chi OR taichi OR "tai ji" OR tai-ji OR taiji OR "qi gong" OR qigong OR qi-gong OR "chi kung" OR "ch i-kung" OR chikung OR ch-i-kung) NEAR/1 (therap* OR treat* OR intervent* OR medicin* OR educat*)):ti,ab
#65 (Wii fit or Kinect):ti,ab
#66 MeSH descriptor: [Animal Assisted Therapy] explode all trees
#67 ((Animal* or pet or pets or dog or dogs or cat or cats*) NEAR/2 (therap* or treat* or intervent*)):ti,ab
#68 MeSH descriptor: [Patient Education as Topic] this term only
#69 MeSH descriptor: [Social Support] this term only
#70 MeSH descriptor: [Self-Help Groups] this term only
#71 MeSH descriptor: [Self Care] this term only
#72 ((Selfhelp* OR self-help* OR ("self" NEXT help*) OR selfcar* OR self-car* OR ("self" NEXT car*) OR self-manag* OR ("self" NEXT manag*) OR support*) NEAR/2 (group* OR car*)):ti,ab
#73 ((patient* OR parent* OR famil* OR relative* OR carer* OR caregiver* OR care-giver* OR spous* OR husband* OR wife* OR wive* OR partner* OR consumer*) NEAR/2 (advis* OR advice* OR counsel* OR educat* OR communicat* OR informat* OR learn* OR lesson* OR librar* OR material* OR need* OR promot* OR resource* OR selfhelp* OR self-help* OR ("self" NEXT help*) OR selfcar* OR self-car* OR ("self"

NEXT car*) OR self-manag* OR ("self" NEXT manag*) OR support* OR teach* OR tool* OR train* OR tutorial*)):ti
#74 MeSH descriptor: [Internet] explode all trees
#75 MeSH descriptor: [Social Networking] explode all trees
#76 MeSH descriptor: [Electronic Mail] explode all trees
#77 MeSH descriptor: [Text Messaging] explode all trees
#78 MeSH descriptor: [Hotlines] explode all trees
#79 Patient Education Handout
#80 MeSH descriptor: [Patient Education Handout] explode all trees
#81 MeSH descriptor: [Pamphlets] explode all trees
#82 ((app OR apps OR blog* OR email* OR e-mail* OR ("e" NEXT mail*) OR facebook OR forum* OR podcast* OR "social media" OR ("social" NEXT network*) OR sms OR ("text" NEXT messag*) OR twitter OR tweet* OR video* OR web* OR smartphone* OR ("smart" NEXT phone*) OR tablet* OR iPad* OR iPhone* OR Skype* OR FaceTime*) NEAR/2 (dementi* OR pseudodementia OR alzheimer* OR alzeimer*)):ti,ab 42
#83 MeSH descriptor: [Bibliotherapy] explode all trees
#84 bibliotherap*:ti,ab
#85 ((book* OR information*) NEAR/2 prescription*):ti
#86 MeSH descriptor: [Self-Help Devices] this term only
#87 ((Assistive* OR automat* OR medicine OR medication OR locate* OR track* OR move* OR alarm* OR safe OR safer OR safety OR communit* OR shut-off OR switch-off) NEAR/2 (technolog* OR device* OR system* OR prompt* OR remind* OR monitor* OR detect* OR sensor* OR dispenser* OR aid OR aids OR solution* OR multicomponent*)):ti,ab
#88 (Clock* OR calendar* OR noticeboard* OR ("notice" NEXT board*) OR notices OR ("sticky" NEXT note*) OR post-it-note* OR ("dossette" NEXT box*) OR ("blister" NEXT pack*)):ti,ab
#89 (telecare* OR ("remote" NEXT monitor*) OR ("video" NEXT confer*) OR ("phone" NEXT block*)):ti,ab
#90 (panic NEAR/2 (button* OR switch* OR alarm*)):ti,ab
#91 ((Environment* OR home* OR (house* NEXT "of" NEXT flat*) OR apartment* OR cottage* OR bungalow*) NEAR/2 (modificat* OR alter* OR adjust* OR adapt* OR revise*)):ti,ab
#92 ((light* NEAR/2 tim*) OR ("water isolation" NEXT device*) OR ("special" NEXT plugs*) OR ("fall" NEXT sensor*)):ti,ab
#93 MeSH descriptor: [Communication] this term only
#94 (Communicat* NEAR/2 (train* OR aid* OR aids OR technolog* OR device* OR system* OR prompt* OR remind*)):ti,ab
#95 ((Adapt* NEAR/2 phone*) OR ("cue" NEXT card*) OR (talk* NEXT mat*)):ti,ab
#96 MeSH descriptor: [Phototherapy] explode all trees
#97 (photo OR photos OR photograph OR photographs OR picture* OR puzzle* OR game* OR (sensor* NEXT stimulat*) OR (mental* NEXT stimulat*) OR ("brain" NEXT train*)):ti,ab
#98 ((Multicomponent* OR multi-component* OR multifacet* OR multi-facet*) NEAR/2 (intervention* OR education* OR support* OR car* OR information*)):ti,ab
#99 MeSH descriptor: [Case Management] explode all trees
#100 ((case NEXT manage*) OR (care NEXT manage)):ti,ab
#101 MeSH descriptor: [Life Change Events] explode all trees
#102 (("life" NEXT review*) OR ("life" NEXT event*) OR ("life" NEXT stor* NEXT book*)):ti,ab 1098
#103 MeSH descriptor: [Vitamins] explode all trees
#104 ((Vitamin* NEXT supplement*) OR (herbal* NEXT remed*)):ti,ab
#105 (Diet* NEAR/2 manage*):ti,ab
#106 Souvenaid*:ti,ab
#107 {OR #23-#106}

#108 #22 AND #107
#109 #22 AND #107 with Publication Year from 2016 to present, in Trials
#110 #22 AND #107 with Cochrane Library publication date from Jan 2016 to present, in Cochrane Reviews

Ovid MEDLINE
1 exp Dementia/
2 (dementi* or pseudodementia).tw.
3 (alzheimer* or alzeimer* or (cortical adj4 sclerosis)).tw.
4 ((encephalopath* or cogniti* or neurocogniti*) adj4 (aids or hiv)).tw.
5 ((aphasi* adj4 (primary or progress*)) or mesulam* or ppa or (ftd adj4 temporal)).tw.
6 (((creutzfeldt or ja?ob*) adj4 (disease or syndrome)) or cjd or (spongiform adj4 encephalopath*) or "corticostriatospinal degeneration" or (pseudosclerosis adj4 spastic)).tw.
7 (binswanger* or ((subcortic* or "sub cortic*" or arterisclerotic) adj4 (encephalopath* or leukoencephalopath*)) or cadasil*).tw.
8 ((kosaka adj2 shibayama) or (neurofibrillary adj1 tangle*) or dntc).tw.
9 (((frontotemporal or (fronto adj temporal) or (corticobasal or (cortico adj basal) or (frontal adj lobe))) adj4 (degenerati* or dysfunction)) or ftld or ftlds or ftd or ftds).tw.
10 ((pick* adj1 (complex or disease* or syndrome)) or (wilhemsen adj1 lynch) or ddpac or (lob* adj4 atroph*)).tw.
11 (huntington* or ((progressive or major or juvenile or hereditary) adj4 chorea)).tw. (19793)
12 (((kluver or kluever) adj4 bu?y) or (("temporal lobectomy" adj4 behavi*) or ("temporal lobe" adj4 dysfunction))).tw.
13 ("lewy bod*" or dlb or lbd or dlbd).tw.
14 ("senile confusion" or "senile psychosis" or senilit*).tw.
15 Tauopathies/
16 tauopath*.tw.
17 cerad.tw.
18 (Posterior adj cortic* adj atroph*).tw.
19 sivd.tw.
20 or/1-19
21 ((Non-pharma* or non pharma*) adj2 (intervent* or therap* or treat* or stimulat* or aid* or techni* or program* or care)).tw.
22 exp Sensory Art Therapies/
23 Complementary Therapies/
24 Counseling/
25 psychotherapy/ or aromatherapy/ or art therapy/ or color therapy/ or dance therapy/ or "imagery (psychotherapy)"/ or music therapy/ or narrative therapy/ or play therapy/
26 ((Sensor* or multisensor* or complement* or alternat* or music* or sound* or acoustic* or audi* or dance* or dancing or play* or art* or touch* or literat* or read* or narrative* or visual* or vision* or light* or colour* or color* or environment* or massage* or aroma* or dawn* or sunshine or sunlight or counsel*) adj1 (intervent* or therap* or stimulat* or aid* or techni* or train* or treat* or counsel* or program*)).tw.
27 (snoezelen* or "senso* environment*" or "senso* room*" or MSE).tw.
28 (Psychotherap* or psycho-therap* or psychoeducat* or psycho-educat*).tw.
29 (Aromatherap* or chromatotherap*).tw.
30 Massage/ or Oils, Volatile/
31 ((Essential or volatile or fragrant) adj1 oil*).tw.
32 exp Cognitive Therapy/ or behavior therapy/

33 ((Cognitive* or cognition* or behaviour* or behavior* or individual*) adj2 (intervent* or therap* or stimulat* or aid* or techni* or train* or skill* or rehab* or treat* or counsel*)).tw.
34 CBT.tw.
35 Holistic Health/
36 ((Validat* or holistic*) adj1 (therap* or method* or techni* or treat* or train* or intervent* or program* or care)).tw.
37 (Memory adj2 (function* or rehab* or therap* or aid* or group* or train* or retraining or support* or stimulation or strateg* or management or managing)).tw.
38 (Reality adj2 orientation).tw.
39 Reminiscence*.tw.
40 Occupational Therapy/
41 ((Occupation* or Occ) adj2 (therap* or treat* or care* or medicine*)).tw.
42 (OT or ergotherap*).tw.
43 language therapy/ or speech therapy {Including Related Terms}
44 ((Speech or talk* or language*) adj2 (intervent* or therap* or stimulat* or aid* or techni* or train* or treat* or counsel* or method* or program*)).tw.
45 "Activities of Daily Living"/
46 (Daily adj2 (life or living) adj2 activi*).tw.
47 ADL.tw.
48 exp *Exercise Therapy/
49 ((Exercis* or kinesiotherap* or kinesiolo* or physical* or sport*) adj1 (therap* or treat* or intervent* or medicin* or educat*)).tw.
50 ((walk* or swim* or jog* or cycling or bicycl* or treadmill* or gym*) adj1 (therap* or treat* or intervent* or medicin* or educat*)).tw.
51 ((Aerobic or yoga or pilates or tai chi or tai-chi or taichi or tai ji or tai-ji or taiji or qi gong or qigong or qi-gong or chi kung or ch i-kung or chikung or ch-i-kung) adj1 (therap* or treat* or intervent* or medicin* or educat*)).tw.
52 (Wii fit or Kinect).tw.
53 Animal Assisted Therapy/
54 ((Animal* or pet or pets or dog or dogs or cat or cats*) adj2 (therap* or treat* or intervent*)).tw.
55 *Patient Education as Topic/
56 *Social Support/
57 Self-Help Groups/
58 Self Care/
59 ((Selfhelp$ or self-help$ or self help$ or selfcar$ or self-car$ or self car$ or self-manag$ or self manag$ or support$) adj2 (group$ or car$)).tw.
60 ((patient$ or parent$ or famil$ or relative$ or carer$ or caregiver$ or care-giver$ or spous$ or husband$ or wife$ or wive$ or partner$ or consumer$) adj2 (advis$ or advice$ or counsel$ or educat$ or communicat$ or informat$ or learn$ or lesson$ or librar$ or material$ or need$ or promot$ or resource$ or selfhelp$ or self-help$ or self help$ or selfcar$ or self-car$ or self car$ or self-manag$ or self manag$ or support$ or teach$ or tool$ or train$ or tutorial$)).ti.
61 exp Internet/
62 Social Networking/
63 Electronic mail/
64 Text messaging/
65 Hotlines/
66 Patient Education Handout/
67 Pamphlets/

68 ((app or apps or blog* or email* or e-mail* or e mail* or facebook or forum* or podcast* or social media or social network* or sms or text messag* or twitter or tweet* or video* or web* or smartphone* or smart phone* or tablet* or iPad* or iPhone* or Skype* or FaceTime*) adj2 (dementi* or pseudodementia or alzheimer* or alzeimer*)).tw
69 Bibliotherapy/
70 bibliotherap$.tw.
71 ((book$ or information$) adj2 prescription$).ti.
72 Self-Help Devices/
73 ((Assistive* or automat* or medicine or medication or locate* or track* or move* or alarm* or safe or safer or safety or communit* or shut-off or switch-off) adj2 (technolog* or device* or system* or prompt* or remind* or monitor* or detect* or sensor* or dispenser* or aid or aids or solution* or multicomponent*)).tw.
74 (Clock* or calendar* or noticeboard* or notice board* or notices or sticky note* or post-it-note* or dossette box* or blister pack*).tw.
75 (telecare* or remote monitor* or video confer* or phone block*).tw.
76 (panic adj2 (button* or switch* or alarm*)).tw.
77 ((Environment* or home* or house* of flat* or apartment* or cottage* or bungalow*) adj2 (modificat* or alter* or adjust* or adapt* or revise*)).tw.
78 ((light* adj2 tim*) or water isolation device* or special plugs* or fall sensor*).tw.
79 *Communication/
80 (Communicat* adj2 (train* or aid* or aids or technolog* or device* or system* or prompt* or remind*)).tw.
81 ((Adapt* adj2 phone*) or cue card* or talk* mat*).tw.
82 exp Phototherapy/
83 (photo or photos or photograph or photographs or picture* or puzzle* or game* or "sensor* stimulat*" or "mental* stimulat*" or "brain train*").tw.
84 ((Multicomponent* or multi-component* or multifacet* or multi-facet*) adj2 (intervention* or education* or support* or car* or information*)).tw.
85 Case Management/
86 ("case manage*" or "care manage").tw.
87 *Life Change Events/
88 ("life review*" or "life event*" or "life stor* book*").tw.
89 exp Vitamins/
90 (Vitamin* supplement* or herbal* remed*).tw. (
91 (Diet* adj2 manage*).tw.
92 Souvenaid*.tw.
93 or/21-92
94 20 and 93
95 Meta-Analysis as Topic/
96 meta analy$.tw.
97 metaanaly$.tw.
98 Meta-Analysis/
99 (systematic adj (review$1 or overview$1)).tw.
100 exp Review Literature as Topic/
101 or/95-100
102 cochrane.ab.
103 embase.ab.
104 (psychlit or psyclit).ab.
105 (psychinfo or psycinfo).ab.

106 (cinahl or cinhal).ab.
107 science citation index.ab.
108 bids.ab.
109 cancerlit.ab.
110 or/102-109
111 reference list$.ab.
112 bibliograph$.ab.
113 hand-search$.ab.
114 relevant journals.ab.
115 manual search$.ab.
116 or/111-115
117 selection criteria.ab.
118 data extraction.ab.
119 117 or 118
120 Review/
121 119 and 120
122 101 or 110 or 116 or 121
123 Comment/ or Letter/ or Editorial/
124 122 not 123
125 94 and 124
126 Animals/ not Humans/
127 125 not 126
128 Randomized Controlled Trials as Topic/
129 randomized controlled trial/
130 Random Allocation/
131 Double Blind Method/
132 Single Blind Method/
133 clinical trial/
134 clinical trial, phase i.pt.
135 clinical trial, phase ii.pt.
136 clinical trial, phase iii.pt.
137 clinical trial, phase iv.pt.
138 controlled clinical trial.pt.
139 randomized controlled trial.pt.
140 multicenter study.pt.
141 clinical trial.pt.
142 exp Clinical Trials as topic/
143 or/128-142
144 (clinical adj trial$).tw.
145 ((singl$ or doubl$ or treb$ or tripl$) adj (blind$3 or mask$3)).tw.
146 PLACEBOS/
147 placebo$.tw.
148 randomly allocated.tw.
149 (allocated adj2 random$).tw.
150 or/144-149
151 143 or 150
152 case report.tw.
153 letter/ or historical article/
154 152 or 153

155 151 not 154
156 94 and 155
157 Animals/ not Humans/
158 156 not 157
159 127 or 158
160 Economics/
161 exp "Costs and Cost Analysis"/
162 Economics, Dental/
163 exp Economics, Hospital/
164 exp Economics, Medical/
165 Economics, Nursing/
166 Economics, Pharmaceutical/
167 Budgets/
168 exp Models, Economic/
169 Markov Chains/
170 Monte Carlo Method/
171 Decision Trees/
172 econom$.tw.
173 cba.tw.
174 cea.tw.
175 cua.tw.
176 markov$.tw.
177 (monte adj carlo).tw.
178 (decision adj3 (tree$ or analys$)).tw.
179 (cost or costs or costing$ or costly or costed).tw.
180 (price$ or pricing$).tw.
181 budget$.tw.
182 expenditure$.tw.
183 (value adj3 (money or monetary)).tw.
184 (pharmacoeconomic$ or (pharmaco adj economic$)).tw.
185 or/160-184
186 Animals/ not Humans/
187 185 not 186
188 94 and 187
189 159 or 188
190 limit 189 to yr="2016 -Current"
191 limit 190 to english language
192 limit 190 to italian
193 191 or 192

Embase

1 exp *Dementia/
2 (dementi* or pseudodementia).tw.
3 (alzheimer* or alzeimer* or (cortical adj4 sclerosis)).tw.
4 ((encephalopath* or cogniti* or neurocogniti*) adj4 (aids or hiv)).tw.
5 ((aphasi* adj4 (primary or progress*)) or mesulam* or ppa or (ftd adj4 temporal)).tw.
6 (((creutzfeldt or ja?ob*) adj4 (disease or syndrome)) or cjd or (spongiform adj4 encephalopath*) or "corticostriatospinal degeneration" or (pseudosclerosis adj4 spastic)).tw.

7 (binswanger* or ((subcortic* or "sub cortic*" or arterisclerotic) adj4 (encephalopath* or leukoencephalopath*)) or cadasil*).tw.
8 ((kosaka adj2 shibayama) or (neurofibrillary adj1 tangle*) or dntc).tw.
9 (((frontotemporal or (fronto adj temporal) or (corticobasal or (cortico adj basal) or (frontal adj lobe))) adj4 (degenerati* or dysfunction)) or ftld or ftlds or ftd or ftds).tw.
10 ((pick* adj1 (complex or disease* or syndrome)) or (wilhemsen adj1 lynch) or ddpac or (lob* adj4 atroph*)).tw.
11 (huntington* or ((progressive or major or juvenile or hereditary) adj4 chorea)).tw.
12 (((kluver or kluever) adj4 bu?y) or (("temporal lobectomy" adj4 behavi*) or ("temporal lobe" adj4 dysfunction))).tw.
13 ("lewy bod*" or dlb or lbd or dlbd).tw.
14 ("senile confusion" or "senile psychosis" or senilit*).tw.
15 *tauopathy/
16 tauopath*.tw.
17 cerad.tw.
18 (Posterior adj cortic* adj atroph*).tw.
19 sivd.tw.
20 or/1-19
21 ((Non-pharma* or non pharma*) adj2 (intervent* or therap* or treat* or stimulat* or aid* or techni* or program* or care)).tw.
22 exp *art therapy/
23 *alternative medicine/
24 *psychotherapy/ or *aromatherapy/ or *color therapy/ or *dance therapy/ or *guided imagery/ or *music therapy/ or *narrative therapy/ or *play therapy/
25 *Counseling/
26 ((Sensor* or multisensor* or complement* or alternat* or music* or sound* or acoustic* or audi* or dance* or dancing or play* or art* or touch* or literat* or read* or narrative* or visual* or vision* or light* or colour* or color* or environment* or massage* or aroma* or dawn* or sunshine or sunlight or counsel*) adj1 (intervent* or therap* or stimulat* or aid* or techni* or train* or treat* or counsel* or program*)).tw.
27 (snoezelen* or "senso* environment*" or "senso* room*").tw. or MSE.ti.
28 (Psychotherap* or psycho-therap* or psychoeducat* or psycho-educat*).tw.
29 (Aromatherap* or chromatotherap*).tw.
30 massage/ or essential oil/
31 ((Essential or volatile or fragrant) adj1 oil*).tw.
32 exp *Cognitive Therapy/ or *behavior therapy/
33 ((Cognitive* or cognition* or behaviour* or behavior* or individual*) adj2 (intervent* or therap* or stimulat* or aid* or techni* or train* or skill* or rehab* or treat* or counsel*)).tw.
34 CBT.ti.
35 Holistic Health.tw.
36 ((Validat* or holistic*) adj1 (therap* or method* or techni* or treat* or train* or intervent* or program* or care)).tw.
37 (Memory adj2 (function* or rehab* or therap* or aid* or group* or train* or retraining or support* or stimulation or strateg* or management or managing)).tw.
38 (Reality adj2 orientation).tw.
39 Reminiscence*.tw.
40 *occupational therapy/
41 ((Occupation* or Occ) adj2 (therap* or treat* or care* or medicine*)).tw.
42 OT.ti. or ergotherap*.tw.
43 *speech therapy/ or *language therapy/

44 ((Speech or talk* or language*) adj2 (intervent* or therap* or stimulat* or aid* or techni* or train* or treat* or counsel* or method* or program*)).tw.
45 *daily life activity/
46 (Daily adj2 (life or living) adj2 activi*).tw.
47 ADL.ti.
48 exp *kinesiotherapy/
49 ((Exercis* or kinesiotherap* or kinesiolo* or physical* or sport*) adj1 (therap* or treat* or intervent* or medicin* or educat*)).tw.
50 ((walk* or swim* or jog* or cycling or bicycl* or treadmill* or gym*) adj1 (therap* or treat* or intervent* or medicin* or educat*)).tw.
51 ((Aerobic or yoga or pilates or tai chi or tai-chi or taichi or tai ji or tai-ji or taiji or qi gong or qigong or qi-gong or chi kung or ch i-kung or chikung or ch-i-kung) adj1 (therap* or treat* or intervent* or medicin* or educat*)).tw.
52 (Wii fit or Kinect).tw.
53 animal assisted therapy/
54 ((Animal* or pet or pets or dog or dogs or cat or cats*) adj2 (therap* or treat* or intervent*)).tw.
55 *patient education/
56 *social support/
57 *self help/
58 *self care/
59 ((Selfhelp$ or self-help$ or self help$ or selfcar$ or self-car$ or self car$ or self-manag$ or self manag$ or support$) adj2 (group$ or car$)).tw.
60 ((patient$ or parent$ or famil$ or relative$ or carer$ or caregiver$ or care-giver$ or spous$ or husband$ or wife$ or wive$ or partner$ or consumer$) adj2 (advis$ or advice$ or counsel$ or educat$ or communicat$ or informat$ or learn$ or lesson$ or librar$ or material$ or need$ or promot$ or resource$ or selfhelp$ or self-help$ or self help$ or selfcar$ or self-car$ or self car$ or self-manag$ or self manag$ or support$ or teach$ or tool$ or train$ or tutorial$)).ti.
61 exp Internet/
62 social network/
63 e-mail/
64 text messaging/
65 hotline/
66 *patient education/
67 Pamphlets.tw.
68 ((app or apps or blog* or email* or e-mail* or e mail* or facebook or forum* or podcast* or social media or social network* or sms or text messag* or twitter or tweet* or video* or web* or smartphone* or smart phone* or tablet* or iPad* or iPhone* or Skype* or FaceTime*) adj2 (dementi* or pseudodementia or alzheimer* or alzeimer*)).tw.
69 bibliotherapy/
70 bibliotherap$.tw.
71 ((book$ or information$) adj2 prescription$).ti.
72 Self-Help Devices/
73 ((Assistive* or automat* or medicine or medication or locate* or track* or move* or alarm* or safe or safer or safety or communit* or shut-off or switch-off) adj2 (technolog* or device* or system* or prompt* or remind* or monitor* or detect* or sensor* or dispenser* or aid or aids or solution* or multicomponent*)).tw.
74 (Clock* or calendar* or noticeboard* or notice board* or notices or sticky note* or post-it-note* or dossette box* or blister pack*).tw.
75 (telecare* or remote monitor* or video confer* or phone block*).tw.

76 (panic adj2 (button* or switch* or alarm*)).tw.
77 ((Environment* or home* or house* of flat* or apartment* or cottage* or bungalow*) adj2 (modificat* or alter* or adjust* or adapt* or revise*)).tw.
78 ((light* adj2 tim*) or water isolation device* or special plugs* or fall sensor*).tw.
79 *interpersonal communication/
80 (Communicat* adj2 (train* or aid* or aids or technolog* or device* or system* or prompt* or remind*)).tw.
81 ((Adapt* adj2 phone*) or cue card* or talk* mat*).tw.
82 exp phototherapy/
83 (photo or photos or photograph or photographs or picture* or puzzle* or game* or "sensor* stimulat*" or "mental* stimulat*" or "brain train*").tw.
84 ((Multicomponent* or multi-component* or multifacet* or multi-facet*) adj2 (intervention* or education* or support* or car* or information*)).tw.
85 *case management/
86 ("case manage*" or "care manage").tw.
87 *life event/
88 ("life review*" or "life event*" or "life stor* book*").tw.
89 exp *vitamin/
90 (Vitamin* supplement* or herbal* remed*).tw.
91 (Diet* adj2 manage*).tw.
92 Souvenaid*.tw.
93 or/21-92
94 20 and 93
95 "meta analysis (topic)"/
96 meta analy$.tw.
97 metaanaly$.tw.
98 meta analysis/
99 "systematic review (topic)"/
100 (systematic adj (review$1 or overview$1)).tw.
101 or/95-100
102 cochrane.ab.
103 embase.ab.
104 (psychlit or psyclit).ab.
105 (psychinfo or psycinfo).ab.
106 (cinahl or cinhal).ab.
107 science citation index.ab.
108 bids.ab.
109 cancerlit.ab.
110 or/102-109
111 reference list$.ab.
112 bibliograph$.ab.
113 hand-search$.ab.
114 relevant journals.ab.
115 manual search$.ab.
116 or/111-115
117 selection criteria.ab.
118 data extraction.ab.
119 117 or 118
120 "review"/

121	119 and 120
122	101 or 110 or 116 or 121
123	editorial/ or letter/
124	122 not 123
125	94 and 124
126	Animals/ not Humans/
127	125 not 126
128	"randomized controlled trial (topic)"/
129	randomized controlled trial/
130	randomization/
131	double blind procedure/
132	single blind procedure/
133	clinical trial/
134	phase 1 clinical trial/
135	phase 2 clinical trial/
136	phase 3 clinical trial/
137	phase 4 clinical trial/
138	randomized controlled trial/
139	multicenter study/
140	"clinical trial (topic)"/
141	or/128-140
142	(clinical adj trial$).tw.
143	((singl$ or doubl$ or treb$ or tripl$) adj (blind$3 or mask$3)).tw.
144	placebo/
145	placebo$.tw.
146	randomly allocated.tw.
147	(allocated adj2 random$).tw.
148	or/142-147
149	141 or 148
150	case report.tw.
151	letter/
152	150 or 151
153	149 not 152
154	94 and 153
155	127 or 154
156	Animals/ not Humans/
157	155 not 156
158	economics/
159	"cost"/
160	exp health economics/
161	pharmacoeconomics/
162	budget/
163	exp economic model/
164	Markov chain/
165	Monte Carlo method/
166	"decision tree"/
167	econom$.tw.
168	cba.tw.
169	cea.tw.

170	cua.tw.
171	markov$.tw.
172	(monte adj carlo).tw.
173	(decision adj3 (tree$ or analys$)).tw.
174	(cost or costs or costing$ or costly or costed).tw.
175	(price$ or pricing$).tw.
176	budget$.tw.
177	expenditure$.tw.
178	(value adj3 (money or monetary)).tw.
179	(pharmacoeconomic$ or (pharmaco adj economic$)).tw.
180	or/158-179
181	94 and 180
182	157 or 181
183	limit 182 to yr="2016 -Current"
184	letter.pt.
185	editorial.pt.
186	note.pt.
187	184 or 185 or 186
188	183 not 187
189	limit 188 to english language
190	limit 188 to italian
191	189 or 190
