# Supplementary material for: The efficacy of cognitive stimulation, cognitive training, and cognitive rehabilitation for people living with dementia: a systematic review and meta-analysis
Source: GeroScience. 2024 Nov 1;47(1):409–44. doi: 10.1007/s11357-024-01400-z (PMC11872969; doi:10.1007/s11357-024-01400-z)
Supplement: Supplementary file 2 — Supplementary file2 (DOCX 1076 KB) [file 11357_2024_1400_MOESM2_ESM.docx]

**Fig. S1** Flow diagram of the methodology approach adopted for the systematic review and meta-analysis


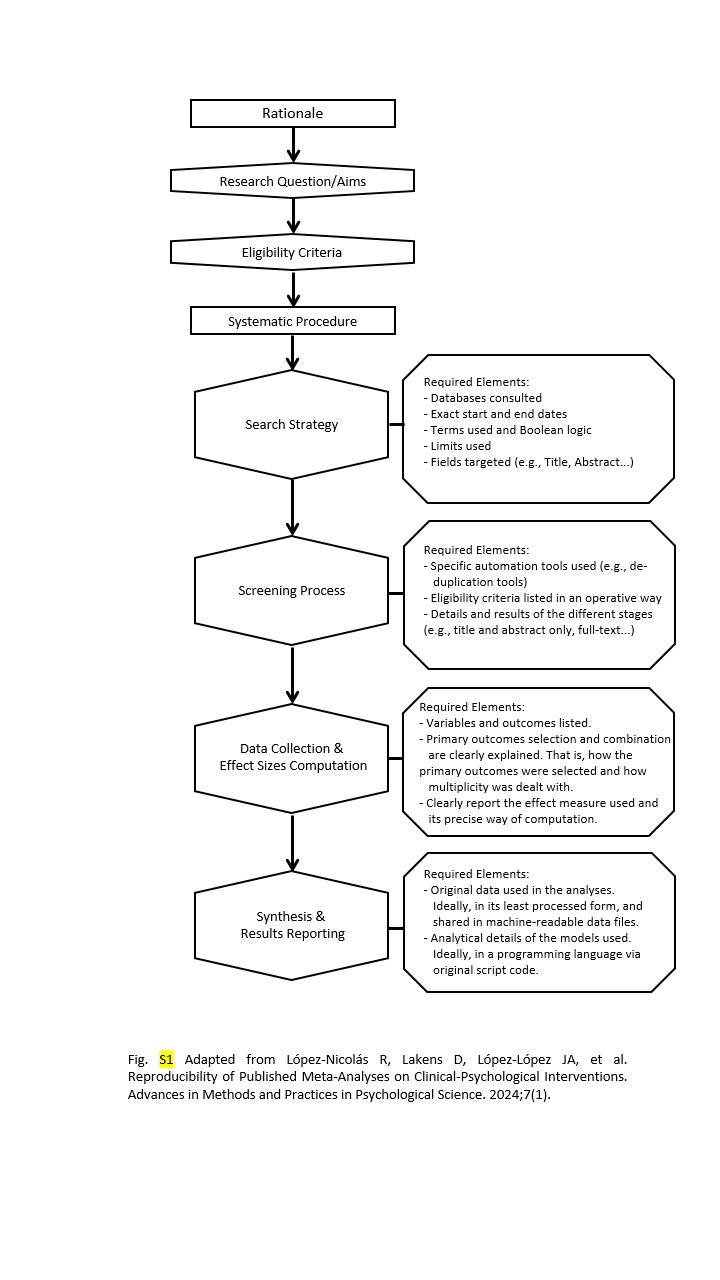


*Note*. Adapted from López-Nicolás et al., 2024.

**Fig. S2a** Quality assessment of studies on Cognitive Stimulation


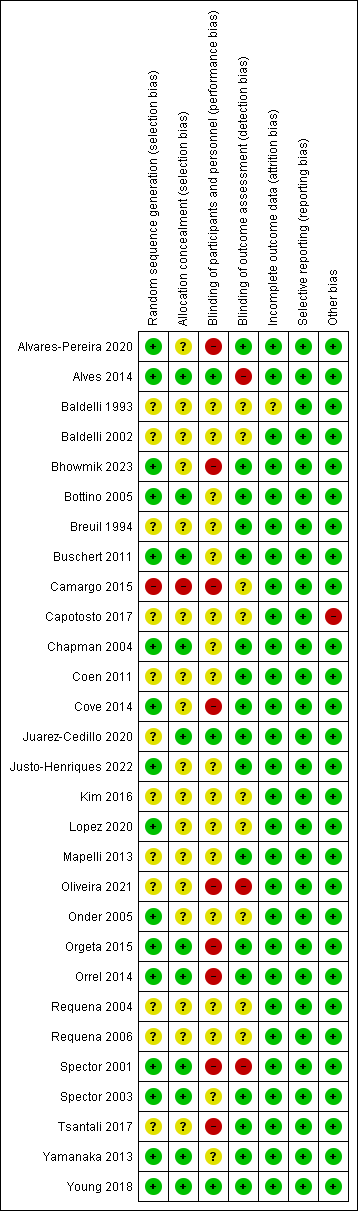


**Fig. S2b** Quality assessment of studies on Cognitive Training


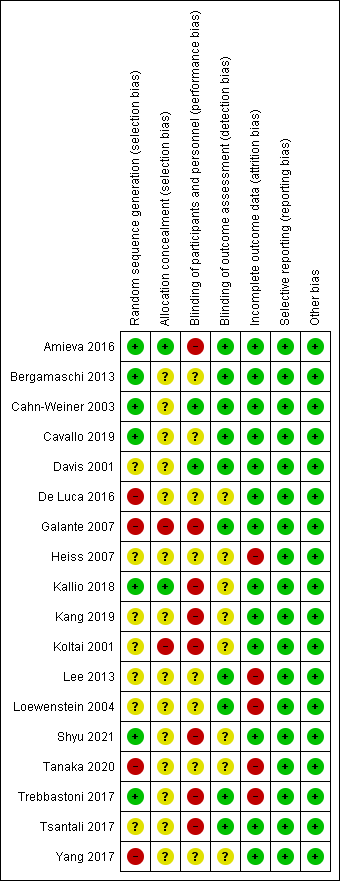


**Fig. S2c** Quality assessment of studies on Cognitive Rehabilitation


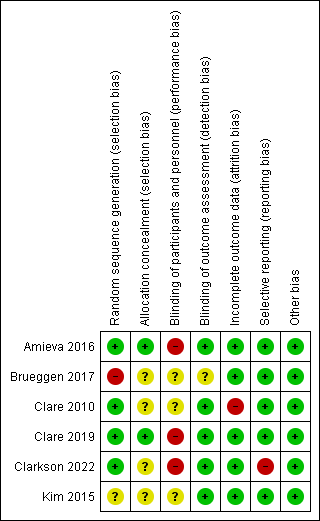


**Fig. S3** Forest plot for group Cognitive Stimulation effect on ADL
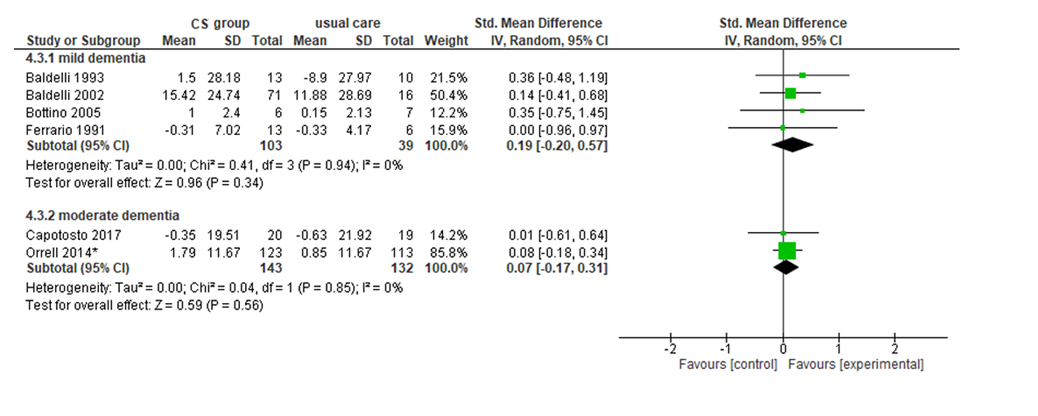
**Note.** ADL: activities of daily living; Chi^2^: heterogeneity test; CI: confidence interval; CS: cognitive stimulation; df: degrees of freedom; I^2^: heterogeneity index; IV: inverse variance method; Random: random effects model; SD: Standard deviation; Z: test for overall standardized mean difference.

*Data calculated by the NICE Guideline working group (NG97).

**Fig. S4** Forest plot for group Cognitive Stimulation effect on quality of life


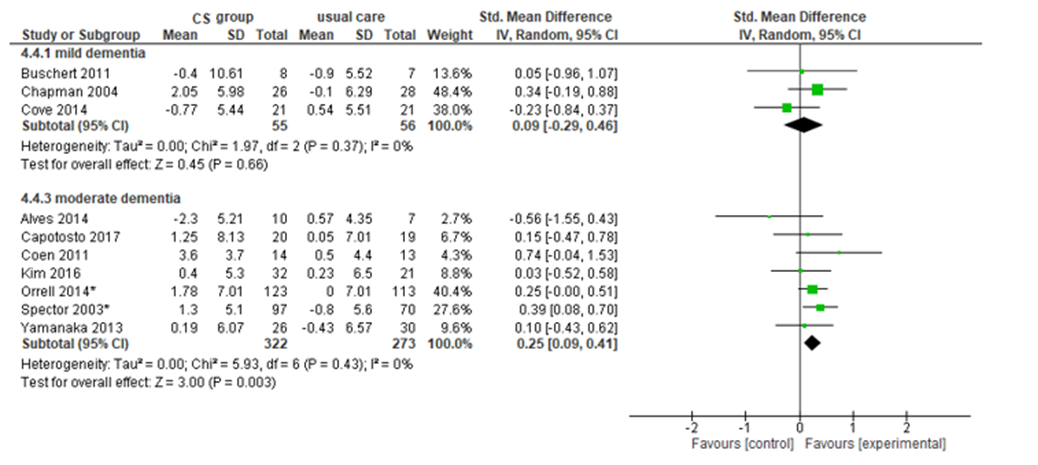
**Note.** Chi^2^: heterogeneity test; CI: confidence interval; CS: cognitive stimulation; df: degrees of freedom; I^2^: heterogeneity index; IV: inverse variance method; Random: random effects model; SD: Standard deviation; Z: test for overall standardized mean difference.

*Data calculated by the NICE Guideline working group (NG97).

**Fig. S5** Forest plot for group Cognitive Training effect on ADL


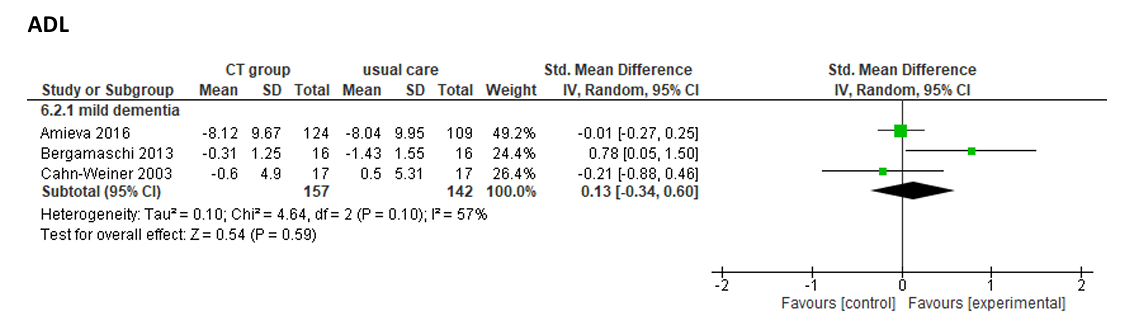
**Note.** ADL: activities of daily living; Chi^2^: heterogeneity test; CI: confidence interval; CT: cognitive training; df: degrees of freedom; I^2^: heterogeneity index; IV: inverse variance method; Random: random effects model; SD: Standard deviation; Z: test for overall standardized mean difference.

**Fig. S6** Forest plot for individual Cognitive Training effect on ADL


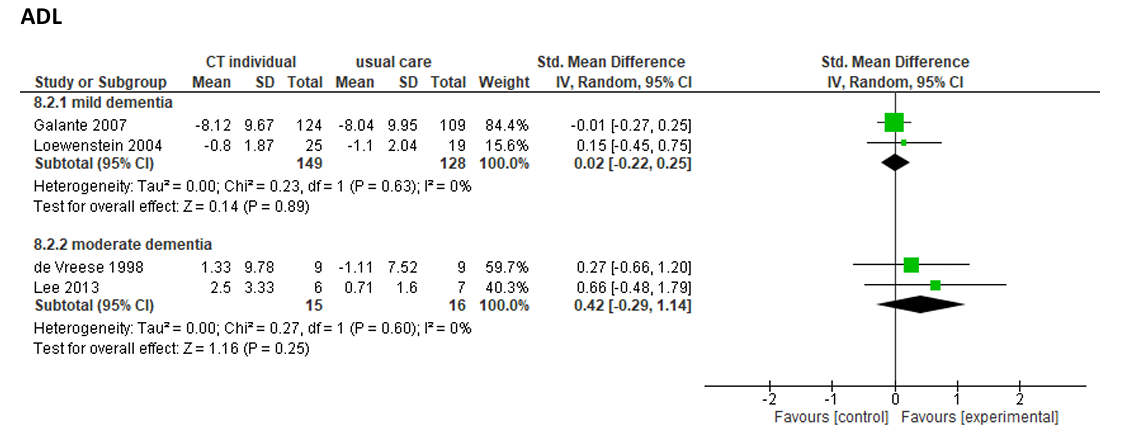
**Note.** ADL: activities of daily living; Chi^2^: heterogeneity test; CI: confidence interval; CT: cognitive training; df: degrees of freedom; I^2^: heterogeneity index; IV: inverse variance method; Random: random effects model; SD: Standard deviation; Z: test for overall standardized mean difference.

**Fig. S7** Forest plot for Cognitive Rehabilitation effect on ADL (scales with higher scores indicating better performance)


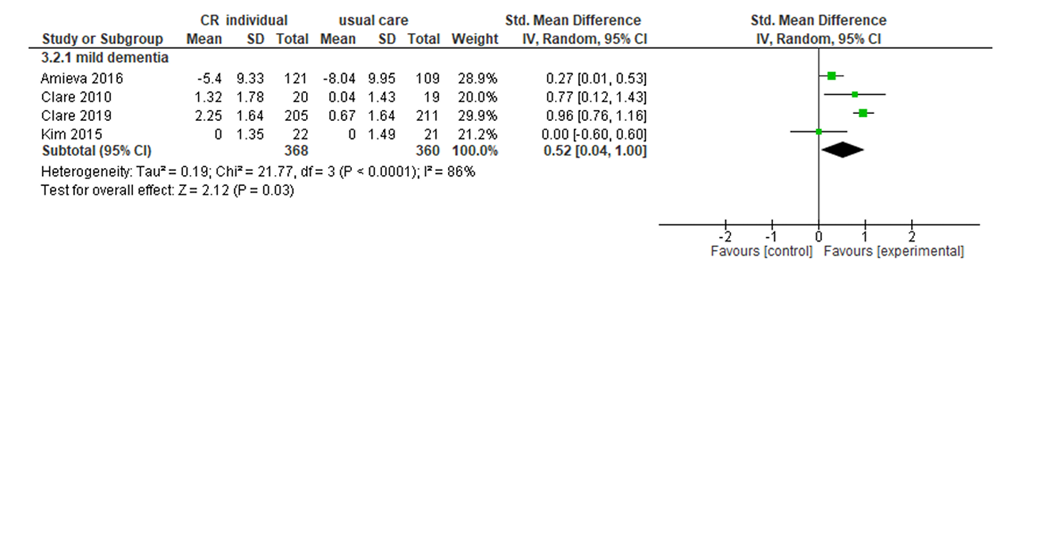
**Note.** ADL: activities of daily living; Chi^2^: heterogeneity test; CI: confidence interval; CR: cognitive rehabilitation; df: degrees of freedom; I^2^: heterogeneity index; IV: inverse variance method; Random: random effects model; SD: Standard deviation; Z: test for overall standardized mean difference.


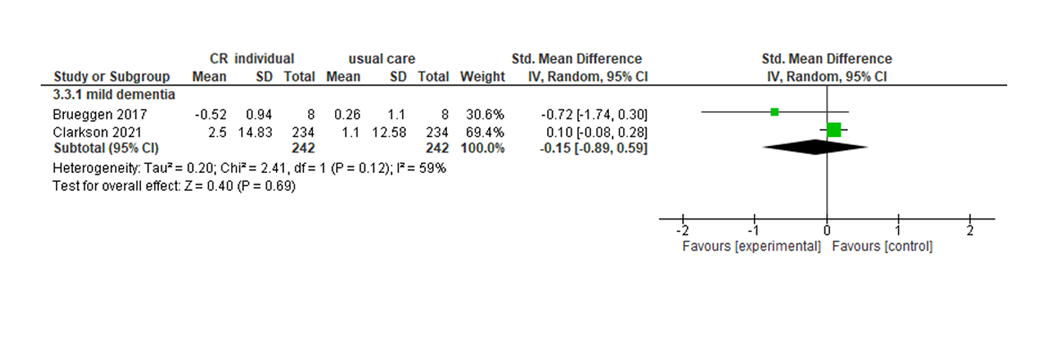
**Fig. S8** Forest plot for cognitive rehabilitation effect on ADL (scales with lower scores indicating better performance)

**Note.** ADL: activities of daily living; Chi^2^: heterogeneity test; CI: confidence interval; CR: cognitive rehabilitation; df: degrees of freedom; I^2^: heterogeneity index; IV: inverse variance method; Random: random effects model; SD: Standard deviation; Z: test for overall standardized mean difference.

**Fig. S9** Forest plot for Cognitive Rehabilitation effect on quality of life


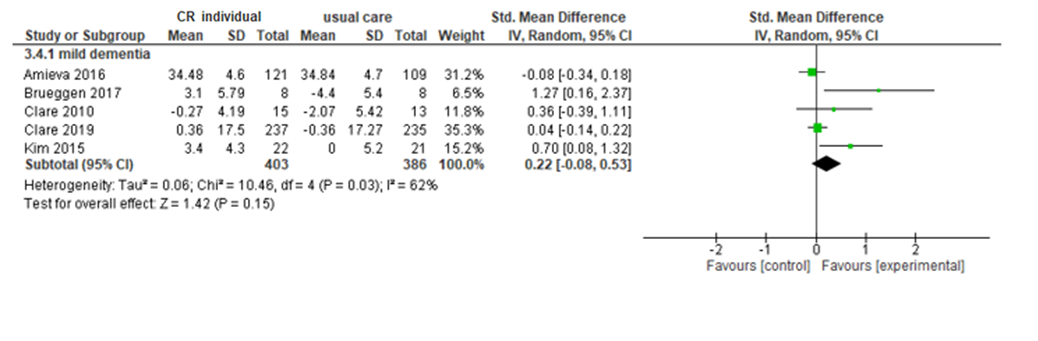


**Note.** Chi^2^: heterogeneity test; CI: confidence interval; CR: cognitive rehabilitation; df: degrees of freedom; I^2^: heterogeneity index; IV: inverse variance method; Random: random effects model; SD: Standard deviation; Z: test for overall standardized mean difference.

**References**

López-Nicolás, R., Lakens, D., López-López, J. A., Rubio-Aparicio, M., Sandoval-Lentisco, A., López-Ibáñez, C., Blázquez-Rincón, D., & Sánchez-Meca, J. (2024). Reproducibility of published meta-analyses on clinical-psychological interventions. *Advances in Methods and Practices in Psychological Science, 7*(1), Article 25152459231202929. [https://doi.org/10.1177/25152459231202929](https://awspntest.apa.org/doi/10.1177/25152459231202929)
