# Supplementary material for: The efficacy of cognitive stimulation, cognitive training, and cognitive rehabilitation for people living with dementia: a systematic review and meta-analysis
Source: GeroScience. 2024 Nov 1;47(1):409–44. doi: 10.1007/s11357-024-01400-z (PMC11872969; doi:10.1007/s11357-024-01400-z)
Supplement: Supplementary file 3 — Supplementary file3 (DOCX 107 KB) [file 11357_2024_1400_MOESM3_ESM.docx]

**Table S1. Characteristics of Included Studies.**

| **Cognitive Stimulation Studies** | | | | | | | | |
| --- | --- | --- | --- | --- | --- | --- | --- | --- |
| **First author (year); Country** | **Criteria for dementia** | **Characteristics of participants** | **Number of participants** | **Type of intervention** | **Definition of usual care** | **Length of treatment** | **Length of follow-up** | **Attrition** - **Lost to follow-up** |
| Alvares-Pereira (2020); Portugal | DSM V | **IG**:  Age: 83.00 (6.67)  M/F: 7/48 **CG** (n= 50):  Age: 84.26 (8.60)  M/F: 7/43 | **Total**: 112  **IG**: 55  **CG**: 57 | Group CST | TAU (i.e. unstructured  occupational activities  such as painting, talking, playing games) | 45 to 60 min. twice a week for 7 weeks (14 sessions in total) | Post  intervention | **Total:** 7  **IG**: 0  **CG**: 7  Lost: 2 died and 5 due to  impossibility of  reassessment (refusal or  discharge) |
| Alves (2014); Portugal | NS | **IG**:  Age: 79.60 (9.06)  M/F: 3/7  MMSE: 17.40 (4.25) **CG**:  Age: 77.71 (12.38)  M/F: 1/6  MMSE: 18.71 (5.02) | **Total**: 17  **IG**: 10  **CG**: 7 | Group CST | TAU: daily routines and  medication | 60 min. 3 times per week for 6 weeks (17 sessions in total) | Post intervention | **Total**: 0 |
| Baldelli (1993); Italy | MMSE > 10 | **Total:**  Age: 84.5 (6.4)  Female: 23 **IG:**  MMSE: 20.1 (4.7) **CG:**  MMSE: 21.3 (5.1) | **Total**: 23  **IG**: 13  **CG**. 10 | Group ROT | TAU | 60 min. 3 times a week for 12 weeks (36 sessions in total) | T1: Post intervention  T2: 3 months after the end  intervention | **Total**: 0 |
| Baldelli (2002); Italy | NS | **IG**:  Age: 79.60 (7.50)  M/F: 21/50  MMSE: 20.77 (2.9) **CG:**  Age: 82.7 (7.00)  M/F: 5/11  MMSE: 20.56 (3.10) | **Total**: 87  **IG**: 71  **CG**: 16 | Group ROT and physical therapy program | Physical therapy program | 60 min. for 5 days a week for 4 weeks (20 sessions in total) | Post intervention | **Total**: 0 |
| Bhowmik (2023); India | DSM V | **IG**:  Age: 66.07 (8.11)  M/F: 17/10 **CG:**  Age: 68.33 (9.33)  M/F: 18/12 | **Total**: 57  **IG**: 27  **CG**: 30 | Group virtual CST | TAU: anti-dementia drug | 45 min. twice a day twice a week for 7 weeks (14 sessions in total) | 1 week post intervention | N.R. |
| Bottino (2005); Brazil | ICD-10 and  NINCDS-ADRDA | **IG**:  Age: 74.67 (6.98)  M/F: 1/5  MMSE: 23.50 (3.27) **CG:**  Age: 72.86 (6.26)  M/F: 3/4  MMSE: 21.29 (3.82)  (all *p* > 0.05) | **Total**: 13  **IG**: 6  **CG**: 7 | Group CSP, rivastigmine  and simultaneously caregivers attended support  group sessions | TAU  (rivastigmine) with 30-min monthly consultations with their doctor | 90 min. once a week for 20 weeks (20 sessions in total) | Post intervention | **Total**: 0 |
| Breuil (1994); France | DSM III | **IG:**  Age: 76.10 (7.1)  M/F: 10/22  MMS: 21 **CG:**  Age: 78.30 (7.00)  M/F: 14/15  MMS: 22 | **Total**: 61  **IG**: 32  **CG**: 29 | Group CSP | No cognitive stimulation | 60 min. for 5 weeks (10 sessions in total) | Post intervention | **Total**: 5  **IG**: 3  **CG**: 2 |
| Buschert (2011); Germany | DSM IV and  NINCDS-ADRDA | **IG:**  Age: 77.3 (7.60)  M/F: 4/4  MMSE: 24.50 (1.60) **CG:**  Age: 74.20 (9.00)  M/F: 3/4  MMSE: 25.30 (1.50) | **Total**: 15  **IG**: 8  **CG**: 7 | Group CSP | Paper-pencil exercises  for self-study, focused on isolated cognitive function | 120 min. weekly for 14 weeks (20 sessions in total) | Post intervention | **Total**: 0 |
| Camargo (2015); Brazil | DSM IV | **IG:**  Age: 80.86 (5.24)  M/F: 1.33:1  MMSE: 22.14 (3.13) **CG**:  Age: 79.43 (7.11)  M/F: 2.5:1  MMSE: 22.71 (2.69) | **Total**: 14  **IG**: 7  **CG**: 7 | Individual ROT and donepezil | TAU (pharmacological therapy) | 30 to 60 min. once a week for 24 weeks (24 sessions in total) | Post intervention | **Total**: 0 |
| Capotosto (2017); Italy | a) MMSE ≥ 14  b) CDR 1 or 2 | **IG:**  Age: 88.25 (5.15)  M/F: 5/15  MMSE: 18.30 (3.14)  **CG:**  Age: 86.52 (5.55)  M/F: 7/12  MMSE: 18.20 (3.63) | **Total**: 39  **IG**: 20  **CG**: 19 | Group CST | Educational activities such as to read articles or  to do creative activities | 45 min. twice a week for 7 weeks (14 sessions in total) | Post intervention | **Total**: 0 |
| Chapman (2004); United States of America | NINCDS-ADRDA | **Total:**  Age (n= 53): 76.38 (7.86)  M/F: 25/29  MMSE (n= 53): 20.87 (3.55) | **Total**: 54  **IG**: 28  **CG**: 26 | Group CSP and donepezil | Donepezil-only | 90 min. once a week for 8 weeks (8 sessions in total) | T1: 2 months after the end  of the intervention  T2: 6 months after the end  of the intervention  T3: 10 months after the end  of the intervention | ND  Missing data from the lost  subjects were processed  through intention-to-treat  analysis |
| Coen (2011); Ireland | MMSE from 10 to 23 | **IG**:  Age: 78.4 (5.00)  M/F: 9/5  MMSE: 16.70 (5.00) **CG**:  Age: 81.30 (6.20)  M/F: 4/9  MMSE (n= 11): 17.10 (5.10) | **Total**: 27  **IG**: 14  **CG**: 13 | Group CST | Usual activities, such as multi-sensory  stimulation programmes, bingo, music, art | 45 min. twice a week for 7 weeks (14 sessions in total) | Post intervention | **Total**: 0 |
| Cove (2014); United Kingdom | DSM IV | **CST + carer training:**  Age: 75.40 (5.56)  M/F: 11/10  MMSE: 22.33 (3.54) **IG:**  Age: 76.80 (6.62)  M/F: 15/9  MMSE: 22.71 (3.76) **CG:**  Age: 77.80 (7.47)  M/F: 10/13  MMSE: 22.91 (3.01) | **Total**: 68  CST + carer training: 21  **IG**: 24  **CG**: 23 | **IG:** Group CST  **CST + carer training**: providing carers the nature and  rationale of CST, introduce essential skills around interacting  with the person they care for, and on implementing activities at home using the guiding principles of CST | wait-list group | 45 min. once a week for 14 weeks (14 sessions in total) | Post intervention | **Total**: 9  **CST + carer training:** 4  **IG**: 3  **CG**: 2  Missing data from the lost  subjects were processed  through intention-to-treat analysis |
| Ferrario (1991)*; NA | NA | **Total:**  Age: 82.5 (5.2)  M/F: 11/8  MMSE range: 18-25 | **Total**: 19 **IG**: 13 **CG**: 6 | Group ROT | Usual care | NA | Post intervention  (6 months) | NA |
| Juarez-Cedillo (2020); Mexico | DSM V and  NINCDS-ADRDA | **IG**:  Age: 77.70 (7.00)  M/F: 16/23  MMSE: 22.40 (0.80) **CG**:  Age: 77.70 (9.30)  M/F: 5/23  MMSE: 22.90 (1.00) | **Total**: 67  **IG**: 39  **CG**: 28 | Group CSP  while simultaneously caregivers were receiving  support from a neuropsychologist | Treatment as usual (drug treatment) | 90 min. twice a week for 48 weeks (96 sessions in total) | T1: Post intervention  T2: 12 months after the end  of intervention  From T1 to T2  Patients and caregivers attended  sessions once a month,  they were given cognitive tasks to carry out at home | **Total**: 17  **IG**: 7  **CG**: 10  Missing data from the lost  subjects were processed  through intention-to-treat analysis |
| Justo-Henriques (2022); Portugal | DSM V | **IG**:  Age: 78.53 (7.82)  M/F: 11/19  MMSE: 22.57 (2.80) **CG:**  Age: 79.21 (7.37)  M/F: 12/17  MMSE: 23.90 (3.53) | **Total**: 59  **IG**: 30  **CG**: 29 | Individual CSP inspired by the principles and  activities used in CST | TAU:  activities based on social interaction, physical activities,  stimulation of personal skills and medication administration | 45 min. once a week for 47 weeks (47 sessions in total) | Post intervention | **Total**: 13  **IG**: 8  **CG**: 5  Main reason of drop out:  discontinued the intervention |
| Kim (2016); Korea | NINCDS-ADRDA | **IG**:  Age: 78.44 (1.00)  M/F: 8/24  MMSE: 18.75 (4.70) **CG** (n= 21):  Age: 78.52 (1.70)  M/F: 8/13  MMSE: 16.95 (6.95) | **Total**: 64  **IG**: 32  **CG**: 32 | Group CSP | TAU (pharmacoterapy only) | 60 min. 5  times a week, for 24 weeks (120 sessions in total) | Post intervention | **Total**: 11  **IG**: 0  **CG**: 11  (irregular medication,  follow-up loss and agreement withdrawal) |
| Lopez (2020); Spain | DSM V | **IG:**  Age: 83.30 (3.71)  M/F: 2/8  MMSE: 18.10 (3.57) **CG:**  Age: 80.50 (6.72)  M/F: 3/7  MMSE: 17.70 (4.24) | **Total**: 20  **IG**: 10  **CG**: 10 | Group CSP and emotional, behavioral and physical therapies | Emotional, behavioral  and physical therapies | 60 min. 3 times a week for 24 weeks (72 sessions in total) | Post intervention | **Total**: 0 |
| Mapelli (2013); Italy | DSM IV-TR | **IG:**  Age: 82.60 (4.85)  MMSE: 20.10 (4.20) **Placebo:**  Age: 84.50 (5.06)  MMSE: 19.70 (3.80) **CG**:  Age: 84.70 (4.42)  MMSE: 18.80 (2.68) | **Total**: 30  **IG**: 10  **Placebo**: 10  **CG**: 10 | Group CSP | **Placebo**:  occupational therapy  **CG**:  TAU  (i.e. read the newspaper, play bingo, sing, pet therapy) | 60 min. 5 times a week or 5 hours once a week for 8 weeks (120 hours in total, 40 sessions maximum) | Post intervention | **Total**: 0 |
| Oliveira (2021); Portugal | NS | **IG**:  Age: 82.60 (5.42)  M/F: 3/7  MMSE: 18.60 (6.48) **CG:**  Age: 84.14 (6.30)  M/F: 2/5  MMSE: 13.00 (7.53) | **Total**: 17  **IG**: 10  **CG**: 7 | Individual computerized CSP with  non-immersive Virtual  Reality with exercises  depicting the IADL | TAU at care units | 45 min. twice a week for 6 weeks (12 sessions in total) | Post-intervention | **Total**: 0 |
| Onder (2005); Italy | NINCDS–ADRA | **IG:**  Age: 75.70 (7.80)  M/F: 21/58  MMSE: 20.20 (3.30) **CG:**  Age: 75.80 (6.30)  M/F: 22/55  MMSE: 19.90 (3.00) | **Total**: 156  **IG**: 79  **CG**: 77 | Individual ROT provided by caregivers  and donepezil | TAU (donepezil only) | 30 min. once a week for 25 weeks (25 sessions in total) | Post intervention | **Total**: 19  **IG**: 9  **CG**: 10 |
| Orgeta (2015); United Kingdom | DSM IV | **IG:**  Age: 78.40 (7.30)  M/F: 97/83  MMSE: 21.12 (4.48) **CG**:  Age: 78.00 (7.70)  M/F: 94/82  MMSE: 21.33 (4.11) | **Total**: 356  **IG**: 180  **CG**: 176 | Individual CST  provided by caregiver | TAU | 30 min. 3 times a week for 25 weeks (75 sessions in total) | Post intervention | **Total**: 83  **IG**: 46  **CG**: 37 |
| Orrell (2014); United Kingdom | DSM IV | **IG:**  Age: 82.70 (7.90)  M/F:43/80  MMSE: 17.80 (5.60) **CG:**  Age: 83.50 (7.20)  M/F: 43/70  MMSE: 17.80 (5.40) | **Total**: 236  **IG**: 123  **CG**: 113 | Group maintenance CST | TAU | 60 min. once a week for 24 weeks (24 sessions in total) | Post intervention | **Total**: 24  **IG**: 17  **CG**: 7 |
| Requena (2004); Spain | DSM III-R  and NINCDS-ADRDA | **CS + drug:** Age: 74.20 (7.81)  M/F: 7/13  MMSE: 22.95 (5.01) **Drug**:  Age: 78.80 (6.62)  M/F: 10/20  MMSE: 21.17 (7.56) **CS:**  Age: 77.00 (7.84)  M/F: 5/13  MMSE: 19.44 (8.18) **CG:** Age: 70.85 (8.12)  M/F: 3/15  MMSE: 19.39 (4.92) | **Total**: 86  **CS+drug**: 20  **Drug**: 30  **CS**: 18  **CG**: 18 | **CS+drug**:  group CSP and donepezil **CS**: group CSP | **Drug**: only donepezil **CG**: no donepezil or cognitive stimulation | 45 min. 5 times a week for 96 weeks (480 sessions in total) | Post intervention | NS |
| Requena (2006); Spain | DSM III-R  and NINCDS-ADRDA | **CS + drug:** Age: 74.20 (7.81)  M/F: 7/13  MMSE: 22.95 (5.01) **Drug:**  Age: 78.80 (6.62)  M/F: 10/20  MMSE: 21.17 (7.56) **CS:**  Age: 77.00 (7.84)  M/F: 5/13  MMSE: 19.44 (8.18) **CG:** Age: 70.85 (8.12)  M/F: 3/15  MMSE: 19.39 (4.92) | **Total**: 86  **CS+drug**: 20  **Drug**: 30  **CS**: 18  **CG**: 18 | **CS+drug**:  group CSP and donepezil **CS**: group CSP | **Drug**: only donepezil **CG**: no donepezil or cognitive stimulation | 45 min. 5 times a week for 96 weeks (480 sessions in total) | Post intervention | NS |
| Spector (2001); United Kingdom | DSM IV | **Total:**  Age: 85.70 (6.70) **IG:**  MMSE: 11.50 (4.40) **CG:**  MMSE: 15.50 (4.40) | **Total**: 35  **IG**: 21  **CG**: 14 | Group CST | TAU | 45 min. twice a week for 8 weeks (15 sessions in total) | Post intervention | **Total**: 8  IG: 4  CG: 4 |
| Spector (2003); United Kingdom | DSM IV | **IG**:  Age: 85.70 (6.20)  M/F: 24/96  MMSE: 14.20 (3.90) **CG**:  Age: 84.7 (7.90)  MMSE: 14.80 (3.80)  M/F: 19/62 | **Total**: 201  **IG**: 115  **CG**: 86 | Group CST | TAU that may include for example games, music or arts | 45 min. twice a week for 7 weeks (14 sessions in total) | Post intervention | **Total**: 34  **IG**: 18  **CG**: 16 |
| Tsantali (2017); Greece | NINCDS-ADRDA and  DSM IV TR | **IG** (n= 17):  Age: 73.30 (4.90)  MMSE: 22.50 (0.90) **CTG** (n= 17):  Age: 73.40 (5.70)  MMSE: 23.20 (1.60) **CG** (n= 21):  Age: 74.20 (5.60)  MMSE: 23.10 (1.40) | **Total**: 63  **IG**: 21  **CTG**: 21  **CG**: 21 | Individual CSP | **CTG:** CT focused on semantic memory,  naming and retrieval ability **CG:** no intervention or other memory assistance | At least 45 min. 3 times a week for 16 weeks (48 sessions in total) | T1: Post intervention  T2: 8 months after the end of the intervention | **Total**: 8  **IG**: 4  **CT**: 4  **CG**: 0 |
| Yamanaka (2013); Japan | MMSE > 10 | **IG**:  Age: 84.12 (5.52)  M/F: 6/20  MMSE: 17.00 (0.83) **CG**:  Age: 83.73 (6.44)  M/F: 6/24  MMSE: 16.87 (0.77) | **Total**: 56  **IG**: 26  **CG**: 30 | Group CST | TAU that included stretching exercises or listening to music, singing, and arts | 45 min. twice a week for 7 weeks (14 sessions in total) | Post intervention | **Total**: 9  **IG**: 3  **CG**: 6  Missing data from the lost  subjects were processed  through intention-to-treat analysis |
| Young (2018); Hong Kong | DSM V | **IG**:  Age: 80.53 (6.26)  M/F: 10/41  MMSE: 20.57 (2.50) **CG**:  Age: 79.86 (6.59)  M/F: 10/40  MMSE: 20.78 (2.09) | **Total**: 101  **IG**: 51  **CG**: 50 | Group CST, combined with thai chi at the end of each group session | TAU that included  interest classes and  recreational activities | 60 min. twice a week for 7 weeks (14 sessions in total) | Post-intervention | **Total**: 9  **IG**: 5  **CG**: 4 |
| **Cognitive Training Studies** | | | | | | | | |
| **First author (year); Country** | **Criteria for dementia** | **Characteristics of participants** | **Number of participants** | **Type of intervention** | **Definition of usual care** | **Length of treatment** | **Length of follow-up** | **Attrition** - **Lost to follow-up** |
| Amieva (2016); France | NINCDS-ADRDA | **CG**: Age: 78.7 (6.5)  M/F: 63/90  MMSE: 21.6 (3.3)  **IG**: Age: 78.5 (7.2)  M/F: 69/ 99  MMSE: 21.5 (3.2)  **RTG**: Age: 78.8 (6.9)  M/F: 61/ 108  MMSE: 21.1 (3.1)  **CR:** Age: 78.7 (6.5)  M/F: 64/92  MMSE: 21.6 (3.0) | **Total**: 653  **CG**: 154  **IG**:170  **RTG**:172  **CRG**:157 | **IG**: group CT (memory, attention, language, EF) and separate sessions arranged in parallel with the caregivers. During these sessions, information upon disease, symptoms, progression, and treatment were delivered by psychologists and medical staff.  **RTG**: different personal themes. During the first 3 months, the caregiver received a telephone contact where they could express a particular difficulty or ask any question. Afterwards, telephone contacts were held every six weeks.  **CRG**: selection of meaningful activities according to the patient; errorless learning if appropriate.  Sessions arranged in parallel with the caregivers. During these sessions, information upon disease, symptoms, progression, and treatment were delivered by psychologists and medical staff. | TAU in AD | 90 min. once a week for 12 weeks, followed by 90 min. every six week for 84 weeks  (26 sessions in total) | T1: Post intervention  T2: post maintenance | **Total**: 181  **CG**: 45  **IG**: 46  **RTG**: 54  **CRG**: 36 |
| Bergamaschi (2013); Italy | DSM V and NINCDS-ADRA | **CG**: Age: 77.72 (5.06)  MMSE: 21.94 (2.01)  **IG:**  Age: 78.19 (5.50)  MMSE: 20.25 (2.95) | **Total**: 32  **CG**: 16  **IG**: 16 | Group paper-and-pencil CT | Non-specific cognitive activities (e.g. reading newspaper, playing group games) | 5 cycles of 120 min. 5 times a week for 4 weeks followed by a 4-weeks break (100 sessions in total) | 1 year | **Total**: 0 |
| Cahn-Weiner (2003); United States of America | NINCDS-ADRDA | **IG**: Age: 77.8 (6.9)  M/F: 9/8  MMSE: 24.3 (2.2)  **CG**: Age: 76.0 (7.7)  M/F: 5/12  MMSE: 25.1 (1.7) | **Total**: 34  **CG**: 17  **IG**: 17 | Group memory training Intervention of the ACTIVE study | Presentation of educational information pertaining to aging and dementia. | 60-75 min. once a week for 6 weeks (6 sessions in total) | 8 weeks after completion of the  training sessions | **Total**: 5  **CG**: 3 **IG**: 2 |
| Cavallo (2019); Italy | NINCDS-ADRDA | **IG**: Age: 79.5 (2.88)  M/F: 13/27  MMSE: 22.65 (1.74)  **CG**: Age: 79.33 (3.83)  M/F: 16/24  MMSE: 23.05 (2.44) | **Total**: 80  **IG**: 40  **CG**: 40 | Individual computerized CT | Free computer use | 30 min. once a week for 12 weeks (12 sessions in total) | 12 months | **Total**: 8  **IG**: 4  **CG**: 4 |
| Davis (2001); United States of America | NINCDS-ADRDA | **IG**:  Age: 68.67 (3.86)  M/F: 10/9  MMSE: 21.84 (4.03)  **CG**:  Age: 72.56 (7.62)  M/F: 6/12  MMSE: 22.78 (4.45) | **Total**: 37  **CG**: 18  **IG**: 19 | Individual CT + home attention exercises | clinic visit (unstructured conversation; reciting overlearned material; watching videotapes) | 30 min./day for 6 days a week for 5 weeks (30 sessions in total) | Post intervention | **Total**: 0 |
| De Luca (2016); Italy | NS | **IG:**  Age: 78.0 (5.4)  M/F: 5/5  MMSE: 24.4 (2.46)  **CG:**  Age: 77.8 (5.3)  M/F: 5/5  MMSE: 24.75 (4.14) | **Total**: 20  **CG**: 10  **IG**: 10 | Individual web-based CT and usual care | Face-to-face interaction; paper-pencil tasks with aids and “100 exercises of cognitive stimulation” book | At least 45 min. 3 times a week for 8 weeks (24 sessions in total) | 8 weeks | **Total**: 0 |
| De Vreese (1999)*; NA | NINCDS-ADRDA | **Total:** Age: NA  M/F: NA  MMSE: 17.2 (3.3) | **Total**: 24 **IG**: NA **CG**: NA | Individual cognitive training (memory, language, executive functioning) facilitated by carer | Usual care | 45 min. twice a week for 12 weeks (24 sessions in total) | Post intervention  (26 weeks) | **Total**: 6 |
| Galante (2007); Italy | NINCDS-ADRDA | **Total:**  Age: 76.0 (6.0)  **IG**:  MMSE: 22.9 (3.1)  **CG**:  MMSE: 23.1 (1.8) | **Total**: 11  **CG**: 4  **IG**: 7 | Individual computer exercises from TNP software. | Semi-structured interview on current affairs; audio-visual material their relatives | 60 min. 3 times a week for 4 weeks (12 sessions in total) | T1: post intervention T2: 3 months after the end of the intervention T3: 9 months after the end of the intervention | **Total**: 0 |
| Heiss (1994); Germany | NINCDS-ADRDA | **IG**:  Age: 65.95 (6.28)  M/F: 9/9  MMSE: 20.55 (4.42)  **SSG:**  Age: 66.63 (10.17)  M/F: 10/7  MMSE: 20.23 (4.10)  **CT-PG:**  Age: 67.18 (8.51)  M/F: 8/9  MMSE: 21.64 (4.55)  **CT-PSG:**  Age: 66.74 (6.93)  M/F: 10/8  MMSE: 20.88 (4.73) | **Total**: 80  **IG**: 18  **SSG**: 17  **CT-PG**: 17  **CT-PSG**: 18 | **IG:** individual CT (memory, perception and movement)  **CT-PG:** individual CT + oral pyritinol 600 mg twice daily  **CT-PSG:** individual CT+ oral phosphatidylserine 200 mg twice daily | Social support (speaking about personal problems and how they managed it; sometimes games used to support conversation) | 60 min. twice a week for 24 weeks (48 sessions in total) | One week after training | **Total**: 10 |
| Kallio (2018); Finland | CDR between 0.5-2 | **IG**:  Age: 82.6 (5.5)  M/F: 26/50  MMSE: 21.0 (4.3)  **CG**:  Age: 83.6 (5.4)  M/F: 15/56  MMSE: 19.9 (3.9) | **Total**: 147  **IG**: 76  **CG**: 71 | Individual paper-and-pencil CT | Usual care (nonspecific social and cognitive group activities) | 45 min. twice a week for 12 weeks (24 sessions in total) | 9 months | **Total**: 30  **IG**: 8  **CG**: 22 |
| Kang (2019); Korea | NINCDS-ADRDA | **CG**:  Age: 68.9 (6.7)  M/F: 16/4  K-MMSE: 22.8 (3.5)  **IG:**  Age: 69.1 (7.9)  M/F: 15/5  K-MMSE: 23.7 (2.8) | **Total**: 40  **IG**: 20  **CG**: 20 | Individual customized paper-based CT | Usual standard clinical care | 60 min. twice a week for 12 weeks (24 sessions in total) | 12 weeks | **Total**: 0 |
| Koltai (2001); United States of America | CDR between 0.5-1.0 | **IG** (n= 14):  Age: 72.9 (6.7)  MMSE: 21.0 (4.3)  **CG** (n= 8):  Age: 73.9 (7.2)  MMSE: 26.6 (2.5) | **Total**: 24  **CG**: 8  **IG**: 16 (8 individual CT; 8 group CT) | Group or individual  memory training | Waiting-list | **Group CT:** 60 min. once a week for 5 weeks (5 sessions in total)  **Individual CT:**  60 min. once a week (mean sessions: 6) | Within 2 weeks post-treatment | **Total**: 2   **IG**: 2  **CG**: 0 |
| Lee (2013); Hong Kong | CDR= 1 | **Total:**  Age: 77.7 (6.07)   **CG**:  M/F: 2/4  MMSE: 17.6 (4.7)  **CELPG:**  M/F: 1/6  MMSE: 15.3 (2.7)  **IG:**  M/F: 3/3  MMSE: 17.0 (3.5) | **Total**: 19  **CG**: 6  **CELP**: 7  **TELP**: 6 | Main training components: memory, strategies to ADL, attention.  **IG:** Individual therapist-led training program (TELP)  **CELPG:**  Individual computer-assisted errorless learning program (CELP) | General cognitively challenging activities | 30 min. twice a week for 6 weeks (6 sessions in total) | 3 months post-intervention | **Total**: 0 |
| Loewenstein (2004);  United States of America | NINCDS-ADRDA and DSM IV | **CG** (n= 19)  Age: 74.74 (7.5)  M/F: 11/8  MMSE: 24.53 (4.5)  **IG** (n=25)  Age: 78.12 (4.3)  M/F: 15/10  MMSE: 23.40 (2.9) | **Total**: 49  **IG**: 28  **CG**: 21 | Individual CT (association, orientation, memory, executive functions) | Individual;  general mental stimulation | 45 min. twice a week for 12-16 weeks (24-32 sessions in total) | 3 months post-intervention | **Total**:  5  **IG**: 3  **CG**: 2 |
| Shyu (2021); Taiwan | NIA-AA | **CG**:  Age: 80 (7)  M/F: 9/6  MMSE: 19 (4)  **IG:**  Age: 82 (5)  M/F: 10/5  MMSE: 22 (3) | **Total**: 30  **IG**: 15  **CG**: 15 | Individual computerized CT program + general written education material | General written education material | 2 cycles of 30 min. once a week for 6 weeks followed by a 4-weeks break (12 sessions in total) | 28 weeks | **Total**:14  **IG**: 7  **CG**: 7 |
| Tanaka (2020);  Japan | MMSE between 5-25 | **CG**:  Age: 84.2 (7.4)  M/F: 7/8  MMSE: 15.6 (5.6)  **IG:**  Age: 88.1 (8.1)  M/F: 6/10  MMSE: 15.4 (5.9) | **Total**: 31  **IG**: 16  **CG**: 15 | Group ROT, physical exercises, CT or cognitive stimulation, cool down + usual care | Daily living assistance and personal rehabilitation | 45 min. twice a week for 8 weeks (16 sessions in total) | 8 weeks | **Total**: 6  **IG**: 1  **CG**: 5 |
| Trebbastoni (2018);  Italy | NINCDS-ADRDA | **IG** (n=45):  Age: 74.26 (6.97)  M/F: 19/26  MMSE: 22.20 (2.37)  **CG** (n=85):  Age: 76.01 (6.46)  M/F: 33/52  MMSE: 22.89 (2.72) | **Total**: 140  **IG**: 54  **CG**: 86 | Group CT | TAU | 75 min. twice a week for 24 weeks (48 sessions in total) | 12 months | **Total**: 10  **IG**: 9  **CG**: 1 |
| Tsantali (2017); Greece | NINCDS-ADRDA and  DSM V TR | **CSG** (n= 17):  Age: 73.30 (4.90)  MMSE: 22.50 (0.90)  **IG** (n= 17):  Age: 73.40 (5.70)  MMSE: 23.20 (1.60)  **CG** (n= 21):  Age: 74.20 (5.60)  MMSE: 23.10 (1.40) | **Total**: 63  **CSG**: 21  **IG**: 21  **CG**: 21 | **IG:** Individual CT  **CSG:** individual cognitive stimulation program | no intervention or other memory assistance | At least 45 min. 3 times a week for 16 weeks (48 sessions in total) | 12 months | **Total**: 8  **CSG**: 4  **IG**: 4  **CG**: 0 |
| Yang (2017);  Korea | NINCDS-ADRDA | **CG**:  Age: 69.9 (8.7)  M/F: 7/3  K-MMSE: 22.7 (3.5)  **IG:**  Age: 71.1 (6.9)  M/F: 7/3  K-MMSE: 23.5 (2.8) | **Total**: 20  **IG**: 10  **CG**: 10 | Individual computer based cognitive program (Brain-Care) | NS | 60 min. twice a week for 12 weeks (24 sessions in total) | 14 weeks | **Total**: 0 |
| **Cognitive Rehabilitation Studies** | | | | | | | | |
| **First author (year); Country** | **Criteria for dementia** | **Characteristics of participants** | **Number of participants** | **Type of intervention** | **Definition of usual care** | **Length of treatment** | **Length of follow-up** | **Attrition** - **Lost to follow-up** |
| Amieva (2016);  France | MMSE between 16-26 | **CG**: Age: 78.7 (6.5)  M/F: 63/90  MMSE: 21.6 (3.3)  **CTG:** Age: 78.5 (7.2)  M/F: 69/ 99  MMSE: 21.5 (3.2)  **RTG:**  Age: 78.8 (6.9)  M/F: 61/ 108  MMSE: 21.1 (3.1)  **IG:**  Age: 78.7 (6.5)  M/F: 64/92  MMSE: 21.6 (3.0) | **Total**: 653  **CG**: 154  **CTG** :170  **RTG**: 172  **IG**: 157 | **IG**: Individual CR (selection of meaningful activities according to the patient; errorless learning if appropriate). **CTG**: group CT (memory, attention, language, EF).  **RTG**: different personal themes (e.g. marriage, working life, ecc). | TAU in AD | 90 min. once a week for 12 weeks, followed by 90 min. every six week for 84 weeks  (26 sessions in total) | T1: Post intervention  T2: post maintenance | **Total**: 181  **CG**: 45  **CTG**: 46  **RTG**: 54  **IG**: 36 |
| Brueggen (2017); Germany | NINCDS-ADRDA | **IG:**  Age: 70.38 (4.54)  M/F: 4/4  MMSE: 21.75 (3.03)  **CG:**  Age: 69.75 (4.03)  M/F=4/4  MMSE: 24 (3.32) | **Total**: 16  **IG**: 8  **CG**: 8 | Individual CORDIAL program. A group-based intervention focused on implementation of daily activities and memory aids. | Standardized CT at home without feedbacks. | 120 min. once a week for 3 months (12 sessions in total) | T0: baseline  T1: 3 months (post-intervention) | **Total**: 2  **IG**: 0  **CG**: 2 |
| Clare (2010); United Kingdom | NINCDS-ADRDA | **IG**:  Age: 76.32 (6.39)  M/F: 9/13  MMSE: 23.14 (3.12)  **RG:**  Age: 77.92 (6.23)  M/F: 10/14  MMSE: 23.33 (2.88)  **CG**:  Age: 78.18 (6.61)  M/F: 9/13  MMSE: 22**.**32 (3.05) | **Total**: 69  **IG**: 23  **RG**: 24  **CG**: 22 | **IG:** Individual goal-oriented CR supported by components addressing practical aids, strategies for learning and maintaining attention and techniques for stress management  **RG:** muscle relaxation and breathing exercises | no contact with researcher | 60 min. once a week for 8 weeks (8 sessions in total) | T1: Post intervention (8 weeks f-u)  T2: 6 months after the end of the intervention (8 months f-u) | **Total**: 13  **IG**: 7  **RG**: 4  **CG**: 2 |
| Clare (2019); United Kingdom | ICD-10 | **CG**:  Age= 78.87 (7.01)  M/F= 124/112  MMSE= 23.75 (3.02)  **IG**:  Age= 78.25 (7.13)  M/F= 124/114  MMSE= 23.89 (3.04) | **Total**: 475  **IG**: 239  **CG**: 236 | Individual goal-oriented CR | TAU (medication, monitoring, general psychosocial support) | 60 min. once a week for 10 weeks over 3 months, followed by 4  60-min. sessions over the next 6 months | 6 months post intervention | **Total**: 48  **IG**: 30  **CG**: 18 |
| Clarkson (2022);  United Kingdom | NS | **IG:**  Age= 79.6 (95%CI 78.7-80.4)  M/F= 112/122  S-MMSE= 22.38 (5.1)  **CG**:  Age= 79.5 (95%CI 78.6-80.4)  M/F= 108/126  S-MMSE= 22.60 (4.7) | **Total** 468  **IG**: 234  **CG**: 234 | Individual CR (range of memory aids, and training and support in their use) and usual care | help from clinic staff, post-diagnostic counselling and advice; specialist follow up as appropriate | 360 min. in 4 weeks | 6 months | **Total**: 112  **IG**: 58  **CG**: 63 |
| Kim (2015);  Korea | MMSE ≥ 18 | **IG:**  Age: 70.40 (7.90)  M/F: 8/14  MMSE: 23.10 (2.10)  **CG:**  Age: 71.40 (8.20)  M/F: 7/14  MMSE: 22.80 (1.80) | **Total**: 43  **IG**: 22  **CG**: 21 | Individual CR (included task of cognitive  training) focused on everyday activities of real life | Unstructured conversation,  questioning with examiner and  watching health-related videos | 60 min. (30 min. individual and 30 min. in group)  once a week for 8 weeks (8 sessions in total) | Post intervention  (8 weeks f-u) | **Total**: 0 |

*Note.* CELPG: Computer-assisted errorless learning program group; CG: control group; CR: Cognitive Rehabilitation; CSP: Cognitive Stimulation Program; CT: Cognitive Training; CT-PG: Cognitive training and pyritinol; CT-PSG: Cognitive training and phosphatidylserine; CSG: Cognitive stimulation group; CST: Cognitive Stimulation Therapy; CTG: Cognitive Training Group; DSM: Diagnostic and Statistical Manual for Mental Disorders; ICD-10: International Statistical Classification of Diseases and Related Health Problems – 10^th^ Edition; IG: Intervention group; MCI: Mild cognitive impairment; MDs: Mean Differences; min.: minutes; MMSE: Mini-Mental State Examination; NA: Not available; NIA-AA: National Institute on Ageing and Alzheimer's Association; NINCDS-ADRDA: National Institute of Neurological and Communicative Disorders and Stroke and the Alzheimer's Disease and Related Disorders Association; NS: Not specified; RG: Relaxation group; ROT: Reality Orientation Therapy; RTG: Reminiscence therapy group; S-MMSE: Standardized Mini-Mental State Examination; SSG: Social support group; TAU: treatment as usual.

* Full-text could not be retrieved.
