# Supplementary material for: The efficacy of cognitive stimulation, cognitive training, and cognitive rehabilitation for people living with dementia: a systematic review and meta-analysis
Source: GeroScience. 2024 Nov 1;47(1):409–44. doi: 10.1007/s11357-024-01400-z (PMC11872969; doi:10.1007/s11357-024-01400-z)
Supplement: Supplementary file 4 — Supplementary file4 (DOCX 31 KB) [file 11357_2024_1400_MOESM4_ESM.docx]

**Table S2. Caregivers’ outcomes in the included studies.**

| **Cognitive Stimulation Studies** | | | | | | | |
| --- | --- | --- | --- | --- | --- | --- | --- |
| **Reference** | **Dementia stage** | **Caregiver** | **N. of caregivers** | **Mean Age (SD)** | **Gender** | **Outcomes measures** | **Narrative results** |
| Alves (2014) | Moderate dementia | Formal caregiver | NR | NR | NR | ZBI | No significant differences. |
| Bhowmik (2023) | NR | Any person engaged in caregiving | NR | NR | NR | ZBI, NPI distress scale | Mean total ZBI score decreased significantly in the CST group, exhibiting less caregiver burden in dementia patients, while ZBI score significantly increased in the control group. |
| Bottino (2005) | Mild dementia | Familiar or professional caregiver | **IG:** 6 **CG:** 7 | NR | NR | HAM-A, MADRS | No significant differences between the groups of caregivers. |
| Onder (2005) | Mild dementia | NR | **IG:** 79 **CG:** 77 | **IG:** 55.1 (13.9) **CG:** 58.4 (12.8) | **IG:**  M/F: 27/52  **CG:**  M/F: 31/46 | HRSD, HRSA, SF–36; CBI | No significant differences. |
| **Cognitive Training Studies** | | | | | | | |
| **Reference** | **Dementia stage** | **Caregiver** | **N. of caregivers** | **Mean**  **Age (SD)** | **Gender** | **Outcomes measures** | **Narrative results** |
| Amieva (2016) | Mild dementia | Family caregiver | **CG:** 154 **IG:** 170 | NR | NR | ZBI, Apathy Inventory | IG did not show statistical significant improvement on ZBI and Apathy Inventory at 3 and 24 months. |
| **Cognitive Rehabilitation Studies** | | | | | | | |
| **Reference** | **Dementia stage** | **Caregiver** | **N. of caregivers** | **Mean Age (SD)** | **Gender** | **Outcomes measures** | **Narrative results** |
| Amieva (2016) | Mild dementia | Family caregiver | **CG:** 154 **IG:** 157 | NR | NR | ZBI, Apathy Inventory | IG did not show statistical significant improvement on ZBI and Apathy Inventory at 3 and 24 months. However, compared to CG, there was a significant improvement on ZBI at 3 months. |
| Brueggen (2017) | Mild dementia | Family caregiver | **IG:** 8  **CG:** 8 | NR | NR | ZBI | No significant differences. |
| Clare (2010) | Mild dementia | Family caregiver | **IG:** 15  **CG:** 13 | 69.9 (12.6) | NR | WHOQOL BREF, GHQ-12, HADS, RSS | Significant differences between CG and IG were observed in the social relationships measured by WHO-QOL BREF. |
| Clare (2019) | Mild dementia | Family caregiver or other supporter | **IG:** 238 **CG:** 236 | **Total**: 68.74 (13.01) **IG:** 68.45 (13.76) **CG:** 69.04 (12.24) | **Total**  M/F: 142/332 **IG:**  M/F: 75/163  **CG:** M/F: 67/169 | EQ 5D, WHOQOL- BREF, RSS | No significant differences. |
| Clarkson (2022) | Mild dementia | Family caregiver | **IG:** 234 **CG:** 234 | **IG:** 65.9 (13.22) **CG:** 66.3 (13.44) | **IG:**  M/F: 73/161  **CG:**  M/F: 73/161 | GHQ-12, SSCQ | No significant differences. |

*Note.* AChE-I: acetylcholinesterase inhibitors; CBI: Caregiver Burden Interview; CG: Control group; EQ 5D: European Quality of Life 5 Dimensions questionnaire Level version; GHQ-12: General Health Questionnaire; HADS: Hospital Anxiety and Depression Scale, HAM-A: Hamilton Anxiety Scale; HRSA: Hamilton Rating Scales for Anxiety; HRSD: Hamilton Rating Scales for Depression; IG: Intervention group; MADRS: Montgomery-Asberg Depression Rating Scale; min.: minutes; NR: Not reported; RSS: Relatives' Stress Scale; SF-36: Medical Outcomes Study 36-item Short-Form General Health Survey; SSCQ: Short Sense of Competence Questionnaire; WHOQOL-BREF: World Health Organisation Quality of Life Instrument—brief version; ZBI: Zarit Burden Interview.
